# Correction: Inhibition of Telomere Recombination by Inactivation of KEOPS Subunit Cgi121 Promotes Cell Longevity

**DOI:** 10.1371/journal.pgen.1005313

**Published:** 2015-06-15

**Authors:** The PLOS Genetics Staff

## Notice of Republication

This article was republished on May 26, 2015, to include details of the editor who handled the submission, which had been omitted due to an error in production. Please download this article again to view the correct version. The originally published, uncorrected article and the republished, corrected article are provided here for reference.

## Supporting Information

S1 File Originally published, uncorrected article. (PDF)

S2 File Republished corrected article. (PDF)
