# Supplementary material for: Inhibition of Telomere Recombination by Inactivation of KEOPS Subunit Cgi121 Promotes Cell Longevity
Source: PLoS Genet. 2015 Mar 30;11(3):e1005071. doi: 10.1371/journal.pgen.1005071 (PMC4378880; doi:10.1371/journal.pgen.1005071)
Supplement: S1 Fig — The full sequences of the three clones of Y’ recombination PCR products in the presence of telomerase. The sequences comprise part of the TRP1 promoter (in green), variable lengths of TG1–3 tracts (including the TG seed in purple and the recombined TG tracts in orange) and the proximal sequences of the Y’ elements (in grey). Clone #1 and #2 contain sequence of Y’ from telomere VIL (Tel06L-YP, in gray), while clone #3 has Y’ sequence from telomere VIIIR (Tel08R-YP, in grey). (PDF) [file pgen.1005071.s001.pdf]

S1 Figure

clone #1

5’ CTTGGCCCTCTCCTTTTCTTTTTTCGACCGAATTAATTCTTAATCG
GCAAAAAAAGAAAAGCTCCGGATCAAGATTGTACGTAAGGTGACA
AGCTATTTTTCAATAAAGAATATCTTCCACTACTGCCATCTGGCGTC
ATAACTGCAAAGTACACATATATTACGATGCTGTTCTATTAAATGCT
TCCTATATTATATATATATAGTAATGTCGTGATCTATGGTGCACTCTCA
GTACAATCTGCTCTGATGGAATTCGTGTGTGGGTGTGGGTGTGGGT
GTGTGGGTGTGTGGGTGTGTGGGTGTGGGTGTGGTGTGGGTGTGG
TGTGGGTGTGGGTGTGTGGGTATATATATGTCACTGTATTGCATGCT
GGATGGTGTTAGACAAGGCCGTAGGGACATATAGCATCTAGGAAG
TAACCTTGTACGAAAATAGGCAATATTTCCTGTTTAGGCGATTGGGA
CGCAGATTTTAGTCCAACGATCTAGCGTCAAGGAATTTTTTTATAGT
GGGACATTGCACCAAGGAAGTAACTTGATACGTCGTGGGTGAATG
GGTCTGTTTTCTTATTCGGCGGGGTAATACATTTTTGGGGGAAGTTT
GTCTGTCTGACGCGCCATATGTAGGTACGCCAAAAAGGGCTCCTCT
ACTTCGAAGCGCGAGGTCGTATACCTAATAAGGAAATGTAATTTAT
AACTTTCTATTATATTGGTCTTTTCGAGAGCGGAAGAAGTTGTAGGC
TAAGCGCAGGCTAAGCGTAGGTCCATGTTTGAAGTATCCAAGAGAA
TATCCACGAATCCTGGTTCTCCTCGACTAAGCAGATAGTTAAGATAC
TGTGCACCATGGAAATTGA **3’**

clone #2

5’ CTTGGCCCTCTCCTTTTCTTTTTTCGACCGAATTAATTCTTAATCG
GCAAAAAAAGAAAAGCTCCGGATCAAGATTGTACGTAAGGTGACA
AGCTATTTTTCAATAAAGAATATCTTCCACTACTGCCATCTGGCGTC
ATAACTGCAAAGTACACATATATTACGATGCTGTTCTATTAAATGCT
TCCTATATTATATATATATAGTAATGTCGTGATCTATGGTGCACTCTCA
GTACAATCTGCTCTGATGGAATTCGTGTGTGGGTGTGGGTGTGGGT
GTGTGGGTGTGTGGGTGTGTGGGTGTGGGTGTGGTGTGGGTGTGG
TGTGGGTGTGGTGGGAATTCTTATACTGTGTGGGTGTGGTGTGTGG
GTGTGTGGGTGTGGTGTGGGTGTGGGTGTGGTGTGGGTGTGGTGT
GTGGGTGTGGTGTGTGTGGGTGTGGTGTGTGGGTGTGGTGTGTGT
GGGTGTGTGGGTATATATATGTCACTGTATTGCATGCTGGATGGTG
TTAGACAAGGCCGTAGGGACATATAGCATCTAGGAAGTAACCTTGT
ACGAAAATAGGCAATATTTCCTGTTTAGGCGATTGGGACGCAGATT
TTAGTCCAACGATCTAGCGTCAAGGAATTTTTTTATAGTGGGACATT
GCACCAAGGAAGTAACTTGATACGTCGTGGGTGAATGGGTCTGTTT
TCTTATTCGGCGGGGTAATACATTTTTGGGGGAAGTTTGTCTGTCTG
ACGCGCCATATGTAGGTACGCCAAAAAGGGCTCCTCTACTTCGAAG
CGCGAGGTCGTATACCTAATAAGGAAATGTAATTTATAACTTTCTAT
TATATTGGTCTTTTCGAGAGCGGAAGAAGTTGTAGGCTAAGCGCAG
GCTAAGCGTAGGTCCATGTTTGAAGTATCCAAGAGAATATCCACGA
ATCCTGGTTCTCCTCGACTAAGCAGATAGTTAAGATACTGTGCACC
ATGGAAATTGA **3’**

clone #3

5’ CTTGGCCCTCTCCTTTTCTTTTTTCGACCGAATTAATTCTTAATCG
GCAAAAAAAGAAAAGCTCCGGATCAAGATTGTACGTAAGGTGACA
AGCTATTTTTCAATAAAGAATATCTTCCACTACTGCCATCTGGCGTC
ATAACTGCAAAGTACACATATATTACGATGCTGTTCTATTAAATGCT
TCCTATATTATATATATATAGTAATGTCGTGATCTATGGTGCACTCTCA
GTACAATCTGCTCTGATGGAATTCGTGTGTGGGTGTGGGTGTGGGT
GTGTGGGTGTGTGGGTGTGTGGGTGTGGGTGTGGTGTGGGTGTGG
TGTGGGTGTGGTGGGAATTCTTATACTGTGTGGGTGTGGTGTGTGT
GGGTGTGTGTGGGTGTGGTGTGTGGGTGTGGTGTGGGTGTGGGTG
TGGGTGTGGTGTGGTGTGTGGGTGTGTGTGTGGGTGTGGTGTGTGT
GTGGGTGTGGTGTGTGTGGGTGTGGTGTGTGGGTGTGGGTGTGGT
GTGTGTGGGTGTGGTGTGTGGGTATATATATGTCACTGTATTGCAT
GCTGGATGGTGTTAGACAAGGCCGTAGGGACATATAGCATCTAGG
AAGTAACCTTGTACGAAAATAGGCAATATTTCCTGTTTAGGCGATTG
TGACGCAGATTTTAGTCCAACGATCTAGCGTCAAGGAATTTTTTTAT
AGTGGGACATTGCACCAAGGAAGTAACTTGATACGTCGTGGGTGA
ATGGGTCTGTCTTCTTATTCGGCGGGGTAATACATTTTTGGGGGAA
GTTTGTCTGTCTGACGCGCCATATGTAGGTACGCCAAAAAGGGCTC
CTCTACTTCGAAGCGCGAGGTCGTATACCTAATAAGGAAATGTAAT
TTATAACTTTTTATTATATTGGTCTTTTCGAGAGCGGAACGTAGGTC
CATGTTTAAAGTATCCAAGAGAATATCCACGAAGCGGCTGAGCAAC
GAACAGAATCCTGGTTCTCCTCGACTAAGCAGATAGTTAAGATACT
GTGCACCATGGAAATTGA **3’**
