# Supplementary figures and images for: Inhibition of Telomere Recombination by Inactivation of KEOPS Subunit Cgi121 Promotes Cell Longevity
Source: PLoS Genet. 2015 Mar 30;11(3):e1005071. doi: 10.1371/journal.pgen.1005071 (PMC4378880; doi:10.1371/journal.pgen.1005071)

## S2 Figure

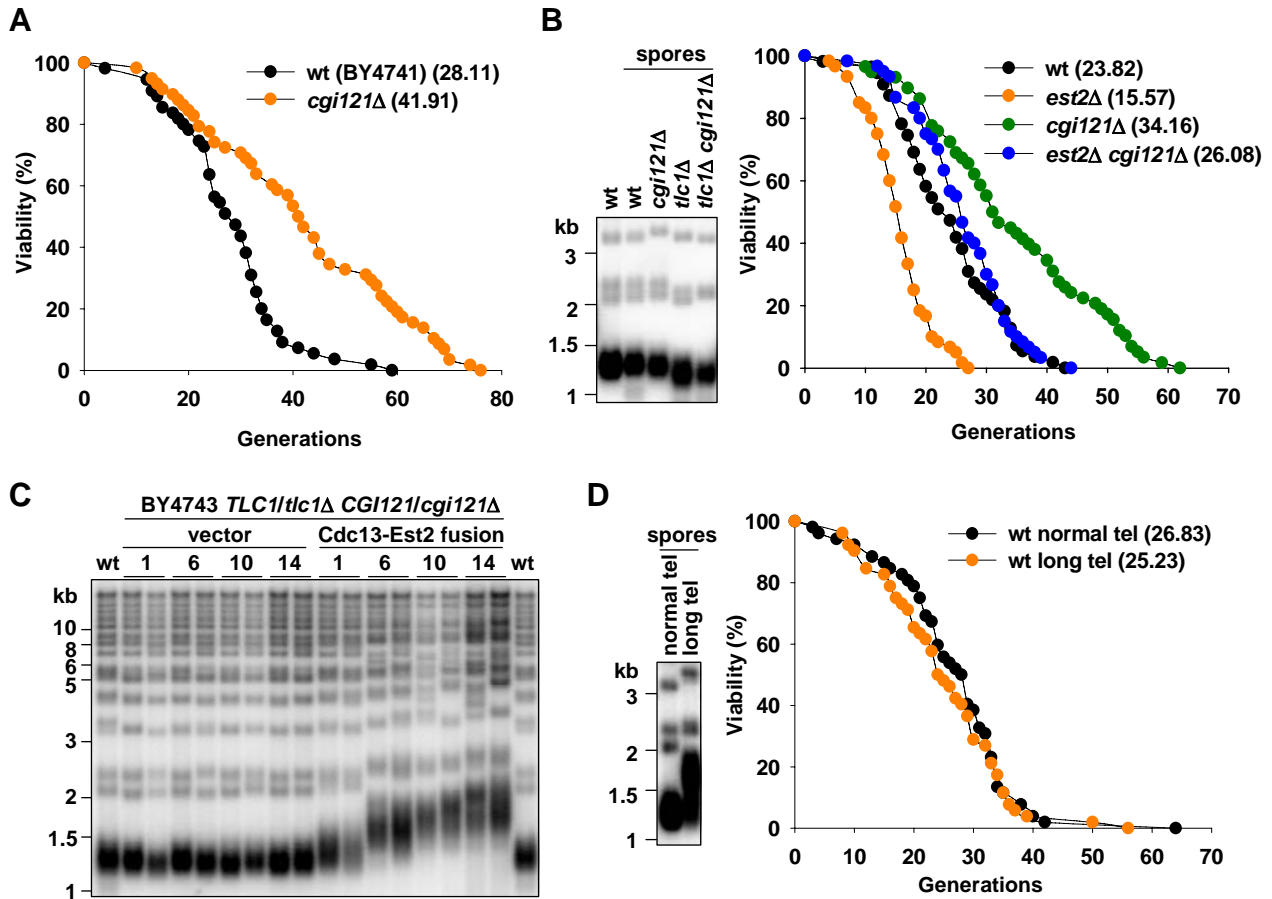

Supplement: S2 Fig — Deletion of CGI121 extends lifespan in both telomerase-positive and pre-senescing telomerase-negative cells. (A) BY4741 cgi121Δ mutant is subjected to lifespan analysis. (B) Spores with different genotypes and the same mating type α were applied to telomere Southern blot (left) and lifespan analysis (right) as in Fig. 3D. (C) After introduction of a plasmid bearing the CDC13-EST2 fusion gene or the vector plasmid as a control, the heterozygous diploid cells were continuously passaged and telomere length was examined at the indicated time point by telomeric Southern blot. The numbers above the lanes indicate the numbers of restreaks. (D) Spores of wild-type cells with normal or long telomeres were subjected to telomere length (left) and lifespan analysis (right). (PDF) [file pgen.1005071.s002.pdf]

S3 Figure

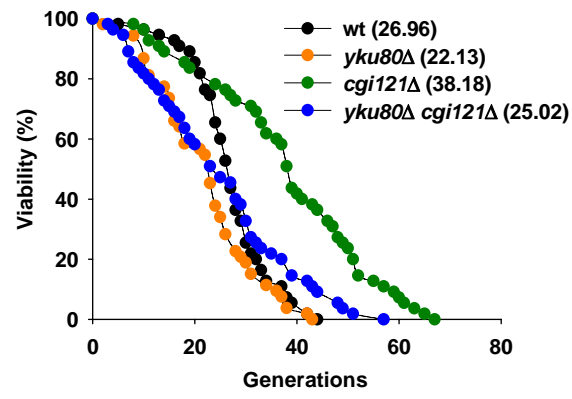

Supplement: S3 Fig — Deletion of CGI121 does not affect lifespan of yku80Δ mutant. Lifespan of yku80Δ and yku80Δ cgi121Δ mutants was examined. (PDF) [file pgen.1005071.s003.pdf]

## S4 Figure

**A**

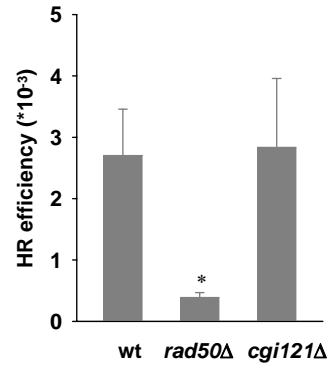

**B**

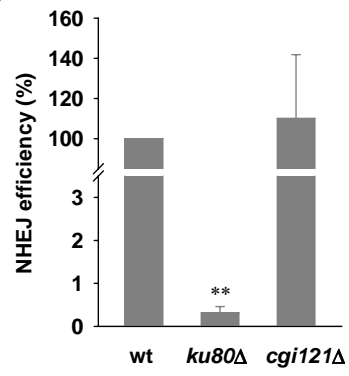

**C**

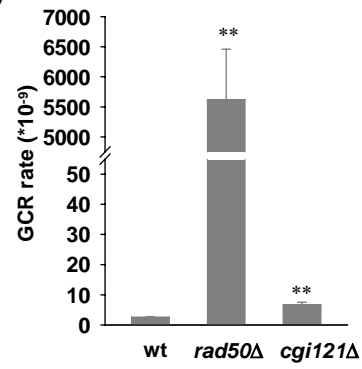

Supplement: S4 Fig — Cgi121 does not regulate HR at other genomic loci, NHEJ or GCR efficiency. (A) HR efficiency at other genomic loci was detected for cgi121Δ mutant (refer to the “Supporting Materials and Methods” session of S1 Text). The error bars indicates the standard deviations. *p < 0.05 and **p < 0.01. (B) NHEJ efficiency of cgi121Δ mutant. The error bars indicates the standard deviations. *p < 0.05 and **p < 0.01. (C) GCR efficiency of cgi121Δ mutant. The error bars indicates the standard deviations. *p < 0.05 and **p < 0.01. (PDF) [file pgen.1005071.s004.pdf]

S5 Figure

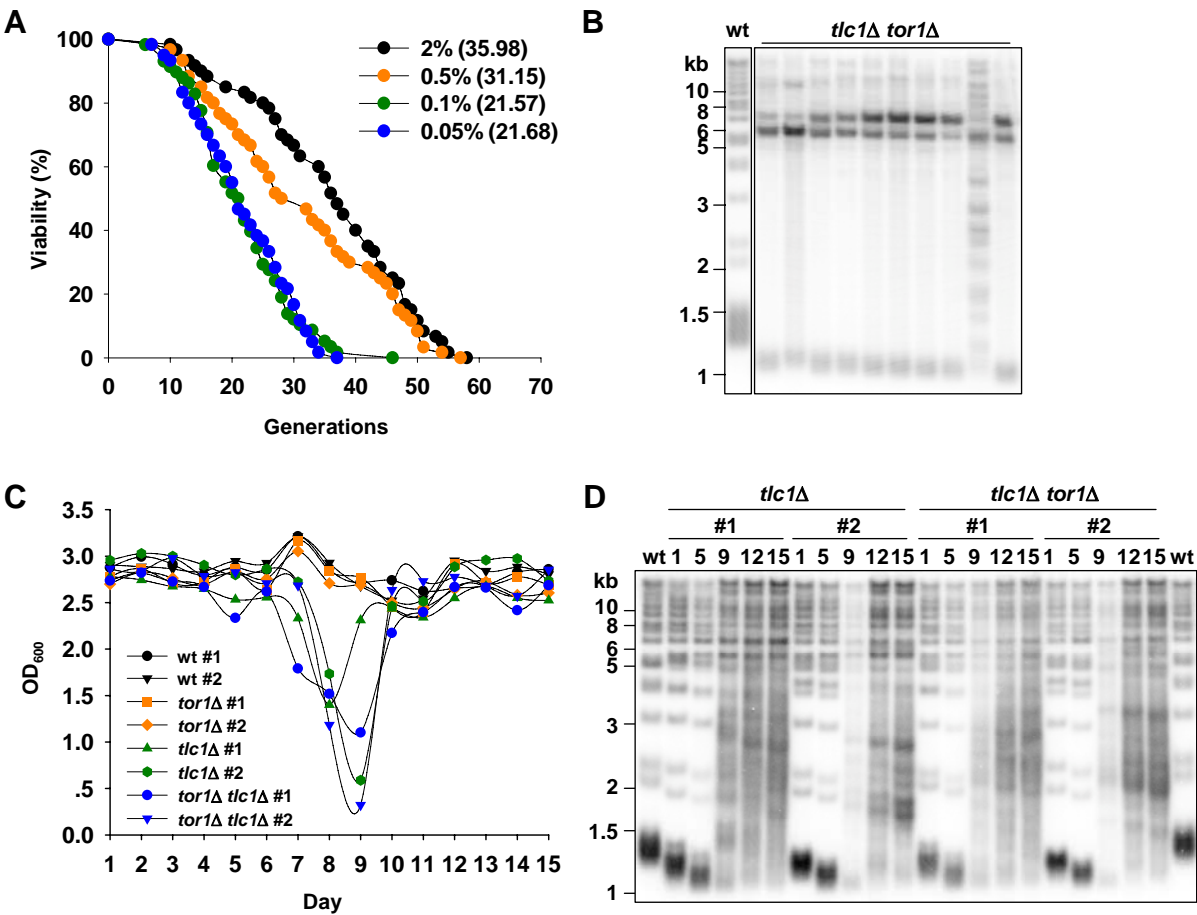

Supplement: S5 Fig — Tor1 does not affect both types of telomere recombination. (A) Lifespan of cgi121Δ mutant under different glucose concentration. (B) Southern blot analysis of 10 clones of tlc1Δ tor1Δ strain obtained by solid medium passage. (C) Growth curve of liquid cultured spores dissected from heterozygous diploid (BY4743 TLC1/tlc1Δ TOR1/tor1Δ). (D) Southern blot analysis of 2 clones each of tlc1Δ and tlc1Δ tor1Δ cells with liquid medium passage. (PDF) [file pgen.1005071.s005.pdf]

## S6 Figure

**A**

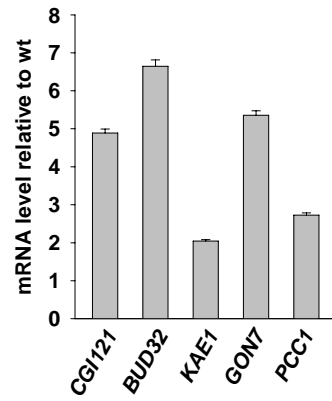

**B**

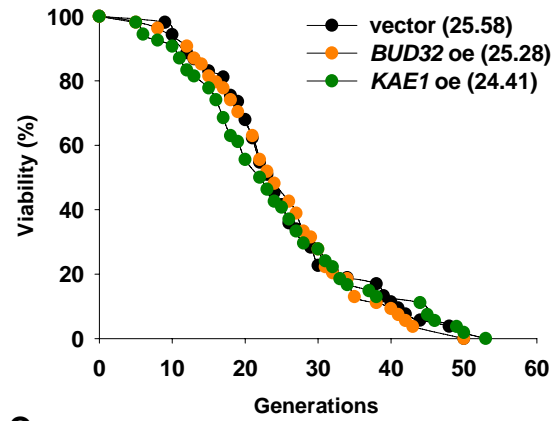

**C**

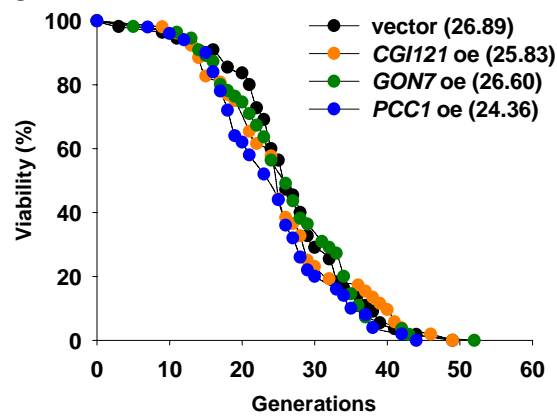

Supplement: S6 Fig — Overexpression of individual KEOPS genes does not affect lifespan. (A) qRT-PCR analysis of the mRNA level of individually overexpressed KEOPS genes. (B) Lifespan analysis of strains overexpressing BUD32 or KAE1. (C) Lifespan analysis of strains overexpressing CGI121, GON7 or PCC1. (PDF) [file pgen.1005071.s006.pdf]
