# Supplementary material for: Inhibition of Telomere Recombination by Inactivation of KEOPS Subunit Cgi121 Promotes Cell Longevity
Source: PLoS Genet. 2015 Mar 30;11(3):e1005071. doi: 10.1371/journal.pgen.1005071 (PMC4378880; doi:10.1371/journal.pgen.1005071)
Supplement: S1 Table — Yeast strains used in this study. (PDF) [file pgen.1005071.s007.pdf]

S1 Table. Yeast strains used in this study.

Figure	Strain	Genotype	Source
Fig. 1	BY4742	*MATα his3Δ1 leu2Δ0 lys2Δ0 ura3Δ0*	Euroscarf
	PJ-092	BY4742 *tlc1Δ::LEU2* Type I	This study
	PJ-093	BY4742 *tlc1Δ::LEU2* Type II	This study
	PJ-094	BY4742 *tlc1Δ::LEU2* Type I *his3Δ1::pRS303-TLC1*	This study
	PJ-095	BY4742 *tlc1Δ::LEU2* Type II *his3Δ1::pRS303-TLC1*	This study
Fig. 2	PJ-096	*MATa-inc ura3-52 lys2-801 ade2-101 trp1Δ63 his3Δ200 leu2Δ1:LEU2-GALHO VII–L::TRP1-TG81-HO site-LYS2 rad52::hisG* pRS316-*RAD52*	Modified from UCC5706 (Diede and Gottschling, 1999)
	PJ-097	*MATa-inc ura3-52 lys2-801 ade2-101 trp1Δ63 his3Δ200 leu2Δ1:LEU2-GALHO VII–L::TRP1-TG81-HO site-LYS2 rad52::hisG* pRS316-*RAD52 rad50Δ::HIS3*	This study
	PJ-098	*MATa-inc ura3-52 lys2-801 ade2-101 trp1Δ63 his3Δ200 leu2Δ1:LEU2-GALHO VII–L::TRP1-TG81-HO site-LYS2 rad52::hisG* pRS316-*RAD52 rad51Δ::ADE2*	This study
	PJ-099	*MATa-inc ura3-52 lys2-801 ade2-101 trp1Δ63 his3Δ200 leu2Δ1:LEU2-GALHO VII–L::TRP1-TG81-HO site-LYS2 rad52::hisG*	This study
	PJ-100	*MATa-inc ura3-52 lys2-801 ade2-101 trp1Δ63 his3Δ200 leu2Δ1:LEU2-GALHO VII–L::TRP1-TG81-HO site-LYS2 rad52::hisG rad50Δ::HIS3 rad51Δ::ADE2*	This study
	PJ-101	*MATa-inc ura3-52 lys2-801 ade2-101 trp1Δ63 his3Δ200 leu2Δ1:LEU2-GALHO VII–L::TRP1-TG81-HO site-LYS2 rad52::hisG* pRS316-*RAD52 tlc1Δ::HIS3*	This study
	PJ-102	*MATa-inc ura3-52 lys2-801 ade2-101 trp1Δ63 his3Δ200 leu2Δ1:LEU2-GALHO VII–L::TRP1-TG0-HO site-LYS2 rad52::hisG* pRS316-*RAD52*	This study
	PJ-103	*MATa-inc ura3-52 lys2-801 ade2-101 trp1Δ63 his3Δ200 leu2Δ1:LEU2-GALHO VII–L::TRP1-TG0-HO site-LYS2 rad52::hisG* pRS316-*RAD52 tlc1Δ::HIS3*	This study
Fig. 3	PJ-096	*MATa-inc ura3-52 lys2-801 ade2-101 trp1Δ63 his3Δ200 leu2Δ1:LEU2-GALHO VII–L::TRP1-TG81-HO site-LYS2 rad52::hisG* pRS316-*RAD52*	Modified from UCC5706 (Diede and Gottschling, 1999)

	PJ-104	*MATa-inc ura3-52 lys2-801 ade2-101 trp1Δ63 his3Δ200 leu2Δ1:LEU2-GALHO VII–L::TRP1-TG0-HO site-LYS2 rad52::hisG* pRS316-*RAD52 cgi121Δ::HIS3*	This study
	PJ-105	BY4741/2 (spore) *tlc1Δ::LEU2*	This study
	PJ-106	BY4741/2 (spore) *tlc1Δ::LEU2 cgi121Δ::URA3*	This study
	BY4742	See Figure 1	Euroscarf
	PJ-107	BY4742 *cgi121Δ::URA3*	This study
	PJ-108	BY4742 (spore)	This study
	PJ-109	BY4742 (spore) *tlc1Δ::LEU2*	This study
	PJ-110	BY4742 (spore) *cgi121Δ::URA3*	This study
	PJ-111	BY4742 (spore) *tlc1Δ::LEU2 cgi121Δ::URA3*	This study
	PJ-112	BY4742 (spore) long telomere	This study
	PJ-113	BY4742 (spore) *tlc1Δ::LEU2* long telomere	This study
	PJ-114	BY4742 (spore) *cgi121Δ::HIS3* long telomere	This study
	PJ-115	BY4742 (spore) *tlc1Δ::LEU2 cgi121Δ::HIS3* long telomere	This study
Fig. 4	PJ-116	BY4742 *ku80Δ::KanMX4 his3Δ1::pRS303*	This study
	PJ-117	BY4742 *ku80Δ::KanMX4 his3Δ1::pRS303-YKU80*	This study
	PJ-118	BY4742 *ku80Δ::KanMX4 his3Δ1::pRS303-yku80-4*	This study
	PJ-119	BY4742 *ku80Δ::KanMX4 his3Δ1::pRS303-YKU80 cgi121Δ::LEU2*	This study
	PJ-120	BY4742 *ku80Δ::KanMX4 his3Δ1::pRS303-yku80-4 cgi121Δ::LEU2*	This study
Fig. 5	BY4742	See Figure 1	Euroscarf
	PJ-121	BY4742 *sir2Δ::KanMX4*	Euroscarf
	PJ-122	BY4742 *fob1Δ::KanMX4*	Euroscarf
	PJ-107	BY4742 *cgi121Δ::URA3*	This study
	PJ-123	BY4742 *fob1Δ::KanMX4 cgi121Δ::URA3*	This study
Fig. 6	BY4742	See Figure 1	Euroscarf
	PJ-124	BY4742 *tor1Δ::KanMX4*	Euroscarf
	PJ-107	BY4742 *cgi121Δ::URA3*	This study
	PJ-125	BY4742 *tor1Δ::KanMX4 cgi121Δ::LEU2*	This study
S2 Figure	BY4741	See Figure 1	Euroscarf
	PJ-126	BY4741 *cgi121Δ::URA3*	This study
	PJ-127	BY4742 (spore)	This study
	PJ-128	BY4742 (spore) *est2Δ::HIS3*	This study
	PJ-129	BY4742 (spore) *cgi121Δ::LEU2*	This study
	PJ-130	BY4742 (spore) *est2Δ::HIS3 cgi121Δ::LEU2*	This study
	BY4743	MATa/α *his3Δ1/his3Δ1 leu2Δ0/leu2Δ0 lys2Δ0/LYS2 MET15/met15Δ0 ura3Δ0/ura3Δ0*	Euroscarf
	PJ-131	BY4743 *TLC1/tlc1Δ::LEU2 CGI121/cgi121Δ::HIS3* pRS316	This study

	PJ-132	BY4743 *TLC1/tlc1Δ::LEU2 CGI121/cgi121Δ::HIS3* pRS316-*CDC13-EST2*	This study
	PJ-133	BY4742 (spore) normal tel	This study
	PJ-134	BY4742 (spore) long tel	This study
S3 Figure	BY4742	See Figure 1	Euroscarf
	PJ-135	BY4742 *ku80Δ::KanMX4*	This study
	PJ-107	BY4742 *cgi121Δ::URA3*	This study
	PJ-136	BY4742 *ku80Δ::KanMX4 cgi121Δ::LEU2*	This study
S4 Figure	HKY660-2B	MATα *leu2-EcoRI::URA3::leu2-BstEII his3-11,15 ade2-1 ura3-1 trp1-1 can1-100*	Klein, 1997
	PJ-137	HKY660-2B *rad50Δ::HIS3*	This study
	PJ-138	HKY660-2B *cgi121Δ::HIS3*	This study
	BY4742	See Figure 1	Euroscarf
	PJ-135	BY4742 *ku80Δ::KanMX4*	This study
	PJ-107	BY4742 *cgi121Δ::URA3*	This study
	PJ-139	BY4742 *hxt13Δ::URA3*	This study
	PJ-140	BY4742 *hxt13Δ::URA3 rad50Δ::KanMX4*	This study
	PJ-141	BY4742 *hxt13Δ::URA3 cgi121Δ::HIS3*	This study
S5 Figure	PJ-107	BY4742 *cgi121Δ::URA3*	This study
	PJ-142	BY4742 (spore)	This study
	PJ-143	BY4742 (spore) *torΔ::HIS3*	This study
	PJ-144	BY4742 (spore) *tlc1Δ::LEU2*	This study
	PJ-145	BY4742 (spore) *tor1Δ::HIS3tlc1Δ::LEU2*	This study
S6 Figure	PJ-146	BY4742 *his3Δ1::pRS303*	This study
	PJ-147	BY4742 *his3Δ1::pRS303-BUD32*	This study
	PJ-148	BY4742 *his3Δ1::pRS303-KAE1*	This study
	PJ-149	BY4742 *his3Δ1::pRS303-CGI121*	This study
	PJ-150	BY4742 *his3Δ1::pRS303-GON7*	This study
	PJ-151	BY4742 *his3Δ1::pRS303-PCC1*	This study
