# Supplementary material for: Inhibition of Telomere Recombination by Inactivation of KEOPS Subunit Cgi121 Promotes Cell Longevity
Source: PLoS Genet. 2015 Mar 30;11(3):e1005071. doi: 10.1371/journal.pgen.1005071 (PMC4378880; doi:10.1371/journal.pgen.1005071)
Supplement: S2 Table — Statistical data of lifespan experiments in this study. (PDF) [file pgen.1005071.s008.pdf]

S2 Table. Statistical data of lifespan experiments in this study.

**Figure**	**Test strain**	**Test LS**	**Test cell #**	**Reference strain**	**Ref LS**	**Ref cell #**	***p* value**
1A	*tlc1*Δ Type I	4.8	60	wt	23.04	53	0.0000
	*tlc1*Δ Type II	13.55	60	wt	23.04	53	0.0000
1C	*tlc1*Δ Type I	4.85	53	wt	27.2	50	0.0000
	*tlc1*Δ Type I *TLC1*	11.62	60	wt	27.2	50	0.0000
	*tlc1*Δ Type I *TLC1*	11.62	60	*tlc1*Δ Type I	4.85	53	0.0000
1D	*tlc1*Δ Type II	14.56	59	wt	27.2	55	0.0000
	*tlc1*Δ Type II *TLC1*	26.17	52	wt	27.2	55	0.4629
	*tlc1*Δ Type II *TLC1*	26.17	52	*tlc1*Δ Type II	14.56	59	0.0000
3C	*cgi121*Δ	33.03	60	wt	21.53	58	0.0000
3D	*tlc1*Δ	21.33	60	wt	25.91	53	0.0185
	*cgi121*Δ	45.26	57	wt	25.91	53	0.0000
	*tlc1*Δ *cgi121*Δ	29.42	60	wt	25.91	53	0.0284
	*tlc1*Δ *cgi121*Δ	29.42	60	*tlc1*Δ	21.33	60	0.0000
	*tlc1*Δ *cgi121*Δ	29.42	60	*cgi121*Δ	45.26	57	0.0000
3E	*tlc1*Δ long tel	26.35	60	wt long tel	25.06	54	0.6074
	*cgi121*Δ long tel	35.71	52	wt long tel	25.06	54	0.0000
	*tlc1*Δ *cgi121*Δ long tel	35.95	60	wt long tel	25.06	54	0.0000
	*tlc1*Δ *cgi121*Δ long tel	35.95	60	*tlc1*Δ long tel	26.35	60	0.0000
	*tlc1*Δ *cgi121*Δ long tel	35.95	60	*cgi121*Δ long tel	35.71	52	0.8657
4A	*yku80*Δ vector	20.46	54	*yku80*Δ *YKU80*	28.02	55	0.0000
	*yku80*Δ *yku80-4*	22.31	54	*yku80*Δ *YKU80*	28.02	55	0.0003
	*yku80*Δ *yku80-4*	22.31	54	*yku80*Δ vector	20.46	54	0.2831
4B	*YKU80* *cgi121*Δ	40.93	55	*YKU80*	27.22	55	0.0000
	yku80-4	20.45	53	*YKU80*	27.22	55	0.0013
	yku80-4 *cgi121*Δ	42.42	55	*YKU80*	27.22	55	0.2925
	yku80-4 *cgi121*Δ	42.42	55	*yku80-4*	20.45	53	0.0000

	yku80-4 cgi121Δ	42.42	55	*YKU80 cgi121Δ*	40.93	55	0.4300
5B	*fob1Δ*	36.05	59	wt	26.35	60	0.0000
	cgi121Δ	35.74	57	wt	26.35	60	0.0003
	fob1Δ cgi121Δ	48.2	59	wt	26.35	60	0.0000
	fob1Δ cgi121Δ	48.2	59	*fob1Δ*	36.05	59	0.0001
	fob1Δ cgi121Δ	48.2	59	*cgi121Δ*	35.74	57	0.0002
6A	*cgi121Δ*	28.88	59	wt	21.2	60	0.0001
	wt + CR	25.3	60	wt	21.2	60	0.0141
	cgi121Δ + CR	20.92	60	wt	21.2	60	0.8277
	cgi121Δ + CR	20.92	60	*cgi121Δ*	28.88	59	0.0000
	cgi121Δ + CR	20.92	60	wt + CR	25.3	60	0.0091
6B	*tor1Δ*	36.49	57	wt	28.12	58	0.0005
	cgi121Δ	42.82	60	wt	28.12	58	0.0000
	fob1Δ cgi121Δ	16.84	58	wt	28.12	58	0.0000
	fob1Δ cgi121Δ	16.84	58	*tor1Δ*	36.49	57	0.0000
	fob1Δ cgi121Δ	16.84	58	*cgi121Δ*	42.82	60	0.0000
S2A	BY4741 *cgi121Δ*	41.91	58	BY4741	28.11	55	0.0000
S2B	*est2Δ*	15.57	60	wt	23.82	55	0.0000
	cgi121Δ	34.16	58	wt	23.82	55	0.0000
	est2Δ cgi121Δ	26.08	60	wt	23.82	55	0.1380
	est2Δ cgi121Δ	26.08	60	*est2Δ*	15.57	60	0.0000
	est2Δ cgi121Δ	26.08	60	*cgi121Δ*	34.16	58	0.0014
S2D	wt long tel	25.23	52	wt normal tel	26.83	52	0.3625
S3	*ku80Δ*	22.13	53	wt	26.96	55	0.0030
	cgi121Δ	38.18	55	wt	26.96	55	0.0000
	ku80Δ cgi121Δ	25.02	55	wt	26.96	55	0.2925
	ku80Δ cgi121Δ	25.02	55	*ku80Δ*	22.13	53	0.3321
	ku80Δ cgi121Δ	25.02	55	*cgi121Δ*	38.18	55	0.0000
S5A	*cgi121Δ* 0.5%	31.15	60	*cgi121Δ* 2%	35.98	60	0.0472
	cgi121Δ 0.1%	21.57	58	*cgi121Δ* 2%	35.98	60	0.0000
	cgi121Δ 0.05%	21.68	60	*cgi121Δ* 2%	35.98	60	0.0000
S6B	*BUD32* oe	25.28	54	vector	25.58	53	0.9207
	KAE1 oe	24.41	54	vector	25.58	53	0.4777
S6C	*CGI121* oe	25.83	52	vector	26.89	55	0.3375
	GON7 oe	26.60	55	vector	26.89	55	0.7972
	PCC1 oe	24.36	50	vector	26.89	55	0.0833
