# Supplementary material for: Inhibition of Telomere Recombination by Inactivation of KEOPS Subunit Cgi121 Promotes Cell Longevity
Source: PLoS Genet. 2015 Mar 30;11(3):e1005071. doi: 10.1371/journal.pgen.1005071 (PMC4378880; doi:10.1371/journal.pgen.1005071)
Supplement: S1 Text — Supporting materials and methods. (PDF) [file pgen.1005071.s009.pdf]

S1 Text. Supporting materials and methods.

General HR and GCR

General HR rate in haploid cells were detected as described previously [78,79]. GCR rate was determined as described previously [80]. *$p < 0.05$ and **$p < 0.01$.

NHEJ

Yeast cells were transformed with equal amount of undigested or EcoRI-linearized plasmid pRS316 and platted to uracil- YC medium. NHEJ efficiency was determined by dividing the number of colonies transformed with digested plasmid by that with undigested plasmid. The efficiency of mutant strains were normalized to that of wild-type cells whose value was set to 1. *$p < 0.05$ and **$p < 0.01$.

Growth curve

Growth curve assay was performed as described previously [35].

qRT-PCR

RNA was extracted and reverse-transcribed, and quantitative Real-time PCR was performed as described previously [81]. At least three biological replicas were done for all the mRNA analysis.

Supporting References

35. Hu Y, Tang HB, Liu NN, Tong XJ, Dang W, et al. (2013) Telomerase-null survivor screening identifies novel telomere recombination regulators. PLoS Genet 9: e1003208.

78. Aguilera A, Klein HL (1988) Genetic control of intrachromosomal recombination in Saccharomyces cerevisiae. I. Isolation and genetic characterization of hyper-recombination mutations. Genetics 119: 779-790.

79. Klein HL (1997) RDH54, a RAD54 homologue in Saccharomyces cerevisiae, is required for mitotic diploid-specific recombination and repair and for meiosis. Genetics 147: 1533-1543.

80. Chen C, Kolodner RD (1999) Gross chromosomal rearrangements in Saccharomyces cerevisiae replication and recombination defective mutants. Nat Genet 23: 81-85.

81. Peng J, Zhou JQ (2012) The tail-module of yeast Mediator complex is required for telomere heterochromatin maintenance. Nucleic Acids Res 40: 581-593.
