# Supplementary material for: Correction: Inhibition of Telomere Recombination by Inactivation of KEOPS Subunit Cgi121 Promotes Cell Longevity
Source: PLoS Genet. 2015 Jun 15;11(6):e1005313. doi: 10.1371/journal.pgen.1005313 (PMC4468270; doi:10.1371/journal.pgen.1005313)
Supplement: S1 File — (PDF) [file pgen.1005313.s001.pdf]

PLOS | GENETICS

RESEARCH ARTICLE

Inhibition of Telomere Recombination by Inactivation of KEOPS Subunit Cgi121 Promotes Cell Longevity

Jing Peng, Ming-Hong He, Yi-Ming Duan, Yu-Ting Liu, Jin-Qiu Zhou*

The State Key Laboratory of Molecular Biology, Shanghai Institute of Biochemistry and Cell Biology, Shanghai Institutes for Biological Sciences, Chinese Academy of Sciences, University of Chinese Academy of Sciences, Shanghai, China

* jqzhou@sibcb.ac.cn

CrossMark
click for updates

OPEN ACCESS

Citation: Peng J, He M-H, Duan Y-M, Liu Y-T, Zhou J-Q (2015) Inhibition of Telomere Recombination by Inactivation of KEOPS Subunit Cgi121 Promotes Cell Longevity. PLoS Genet 11(3): e1005071. doi:10.1371/journal.pgen.1005071

Received: October 14, 2014

Accepted: February 13, 2015

Published: March 30, 2015

Copyright: © 2015 Peng et al. This is an open access article distributed under the terms of the Creative Commons Attribution License, which permits unrestricted use, distribution, and reproduction in any medium, provided the original author and source are credited.

Data Availability Statement: All relevant data are within the paper and its Supporting Information files.

Funding: This work was supported by grants from National Natural Science Foundation of China [NSFC31230040/31221001] (http://www.nsfc.gov.cn/) and Ministry of Science and Technology of the People's Republic of China [2013CB910403/2011CB966301 to JQZ (http://www.most.gov.cn/eng/). The funders had no role in study design, data collection and analysis, decision to publish, or preparation of the manuscript.

Competing Interests: The authors have declared that no competing interests exist.

Abstract

DNA double strand break (DSB) is one of the major damages that cause genome instability and cellular aging. The homologous recombination (HR)-mediated repair of DSBs plays an essential role in assurance of genome stability and cell longevity. Telomeres resemble DSBs and are competent for HR. Here we show that in budding yeast *Saccharomyces cerevisiae* telomere recombination elicits genome instability and accelerates cellular aging. Inactivation of KEOPS subunit Cgi121 specifically inhibits telomere recombination, and significantly extends cell longevity in both telomerase-positive and pre-senescing telomerase-negative cells. Deletion of *CGI121* in the short-lived *yku80*^{tel} mutant restores lifespan to *cgi121Δ* level, supporting the function of Cgi121 in telomeric single-stranded DNA generation and thus in promotion of telomere recombination. Strikingly, inhibition of telomere recombination is able to further slow down the aging process in long-lived *fob1Δ* cells, in which rDNA recombination is restrained. Our study indicates that HR activity at telomeres interferes with telomerase to pose a negative impact on cellular longevity.

Author Summary

Aging is a general biological process among the living organisms which is affected by environmental stimuli but also genetically controlled. Genome instability is one of the aging hallmarks and has long been implicated as one of the main causal factors in aging. DNA double strand breaks (DSBs) are the most deleterious DNA damages that cause genome instability. To counteract DNA damage of DSBs and maintain high level of genome integrity, cells have evolved powerful repair systems such as homologous recombination (HR). HR is crucial for DNA repair and genome integrity maintenance, and is generally believed to be essential for assurance of cell longevity. Telomeres, the physical ends of eukaryotic linear chromosomes, are preferentially elongated by telomerase, a specialized reverse transcriptase, in most cases. However, due to the resemblance of telomeres to DSBs, HR can not be eliminated but rather readily takes place on telomeres, even in the presence of telomerase. Here we show that HR at yeast telomeres elicits genome instability and accelerates

cellular aging. Inactivation of the evolutionarily conserved KEOPS complex subunit Cgi121 specifically inhibits telomere HR and results in extremely long lifespan, indicating a dark side of HR in longevity regulation.

Introduction

Aging is generally defined as the time-dependent functional decline and increased mortality in most living organisms. Although aging appears to be a natural process, increasing evidence indicates that aging is genetically controlled. In order to elucidate how aging is influenced by intrinsic cellular traits, researchers have developed and employed various model organisms including yeast, worm, fly, fish, mouse and monkey to study the pathways that affect aging. The single-cell organism, budding yeast *Saccharomyces cerevisiae* represents a widely used tool for aging study [1,2,3]. A single yeast mother cell can only generate a limited number of daughter cells before its mitotic arrest [4]. This aging-associated phenotype is called replicative aging [5]. The organismal aging for multicellular species is likely (or at least partially) to be attributed to cellular aging in their corresponding organs and/or tissues.

The genome, which carries the genetic information of a cell, is continuously threatened by exogenous damages, as well as by endogenous threats such as DNA replication errors [6]. Genome instability is one of the aging hallmarks, and has long been implicated as one of the main causal factors in aging [7,8]. DNA damage (e.g. double strand break, DSB) is one of the major causes for genome instability. When the repair pathways are not efficient enough to cope with a given level of damage, cells may undergo cell cycle arrest, cellular senescence and cell death. For example, the Werner syndrome and Bloom syndrome, two typical progeroid syndromes, are respectively caused by defective helicases WRN and BLM, which are involved in DNA repair [9]. The cells from both syndromes show increased DNA damage accumulation [9]. Consistently, the deficiency in yeast Sgs1 helicase, the homologue of human WRN and BLM, also results in genome instability, such as enhancement of rDNA recombination and fragmentation of nucleolus, and leads to premature cellular aging [10]. To maintain genome stability, genome maintenance pathways have emerged during evolution, and function in longevity assurance. For example, homologous recombination (HR) and non-homologous end joining (NHEJ) pathways have been evolved to repair the most deleterious DNA damages, the DNA double strand breaks (DSBs). Accordingly, mutation of yeast DSB repair genes, such as *RAD50*, *RAD51*, *RAD57* and *RAD52*, greatly reduces yeast replicative lifespan [11].

Telomeres are the physical ends of eukaryotic linear chromosomes, and are crucial for genome integrity and stability [12]. Although telomeres may look like DSBs as the chromosomal ends, they are distinguished by the specialized architecture, consisting of repetitive guanine-rich DNA bound by telomere-specific proteins. The yeast telomeric DNA consists of $\sim$350 bp of $TG_{1-3}/C_{1-3}A$ repeats, and the G strand extends beyond its complementary strand to form a single-stranded overhang, called the G-overhang [12,13,14]. The telomerase complex, which consists of the catalytic subunit Est2, the template RNA moiety Tlc1, and two accessory subunits Est1 and Est3, is responsible for telomeric G-strand elongation, as well as telomere protection [15,16,17,18]. When telomerase is inactivated, telomeres keep shortening and most cells undergo critically short telomere-triggered cell cycle arrest, a process termed "replicative senescence" [16,19,20]. "Replicative senescence" is usually considered to be different from "replicative aging" as the former is largely attributed to critically short telomeres. Although telomeres are well protected and excluded from DSB repair at most of the time, yet there are several traits that make telomeres highly prone to be recombined. Firstly, all the telomeres are

much alike in their repetitive sequences which could be favorable substrates for homologous recombination activities. Additionally, quite a few proteins (or protein complexes) involved in DNA repair pathways also bind and function at telomeres [14]. The yKu70/80 heterodimer, which is required for NHEJ [21,22], is indispensable for telomere protection, telomerase recruitment and telomeric heterochromatin maintenance [23,24,25,26,27,28]. Mre11/Rad50/Xrs2 heterotrimer, DNA helicase Sgs1 and endonuclease Sae2, which are critical for resection of the ends of DSBs in HR-mediated DSB repair, are involved in telomere 5' end resection after DNA replication [29,30,31]. Moreover, the 3' overhang generated by telomere end resection could be perceived as intermediates of DSB, as the strand invasion step requires the ssDNA [32]. Thus, considering all the traits of telomeres mentioned above, we propose that recombination activity in yeast might covet telomeres and interfere with telomerase to elicit genome instability under physiological conditions, and thereby affect cellular longevity.

The evolutionary conserved KEOPS complex which consists of five subunits, i.e. Cgi121, Bud32, Kae1, Gon7 and Pcc1 in yeast, was first identified as a telomere regulator [33,34]. Deletion of *CGI121* or *BUD32* reduces single-stranded telomeric DNA accumulated in *cdc13-1* cells, and suppresses the temperature sensitivity of *cdc13-1* mutant grown at 28°C [33], indicating that loss of Cgi121 or Bud32 limits the amount of ssDNA generated at uncapped telomeres. Moreover, deletion of any subunit of KEOPS complex results in defect in telomere recombination [35], suggesting that KEOPS complex promotes telomeric TG_{1-3} tracts recombination. In addition to telomere regulation, KEOPS complex also participates in tRNA modification (t6A) [36,37]. Interestingly, the Cgi121 subunit of the KEOPS complex is indispensable for both telomere length regulation and recombination, but not required for tRNA modification [33,37]. We therefore exploited the separation-of-function subunit Cgi121 to dissect the functions of KEOPS in telomere recombination from those in tRNA modification. Our data presented in the current work indicate that activation of telomere recombination accelerates cellular aging, and attenuation of telomere recombination, e.g. by inactivation of Cgi121, promotes cell longevity.

Results

Telomerase-null survivors have shorter replicative lifespan than telomerase-proficient cells

When telomerase is inactivated by deletion of a gene encoding telomerase subunit (Est1, Est2, Est3 or Tlc1), telomeres gradually shorten, and most cells undergo senescence after 75 to 100 generations due to the critical short telomeres [16,19,20]. Because critical short or deprotected telomeres are highly recombinogenic, a small percentage of the telomerase-null cell can overcome the crisis by using homologous recombination to maintain their telomeres. These so-called "survivors" either have telomeres with amplified subtelomeric Y' sequence and short TG_{1-3} tracts (the Type I survivors) [38], or harbor long heterogeneous TG_{1-3} sequence (Type II survivors) [39].

In order to address the effect of telomere recombination on replicative aging, we examined lifespan of the survivor cells. After deletion of telomerase subunit *TLC1*, cells were serially passaged in solid or liquid medium to obtain Type I and Type II survivors respectively (Fig. 1A, left) [35]. Lifespan assay was performed with both types of survivors. The results showed that both the Type I and Type II survivors have much shorter lifespan than wild-type cells, and the lifespan of Type I survivors is extremely short (Fig. 1A, right). This result is consistent with our previous data that *est2*Δ Type II survivors have shorter lifespan [40].

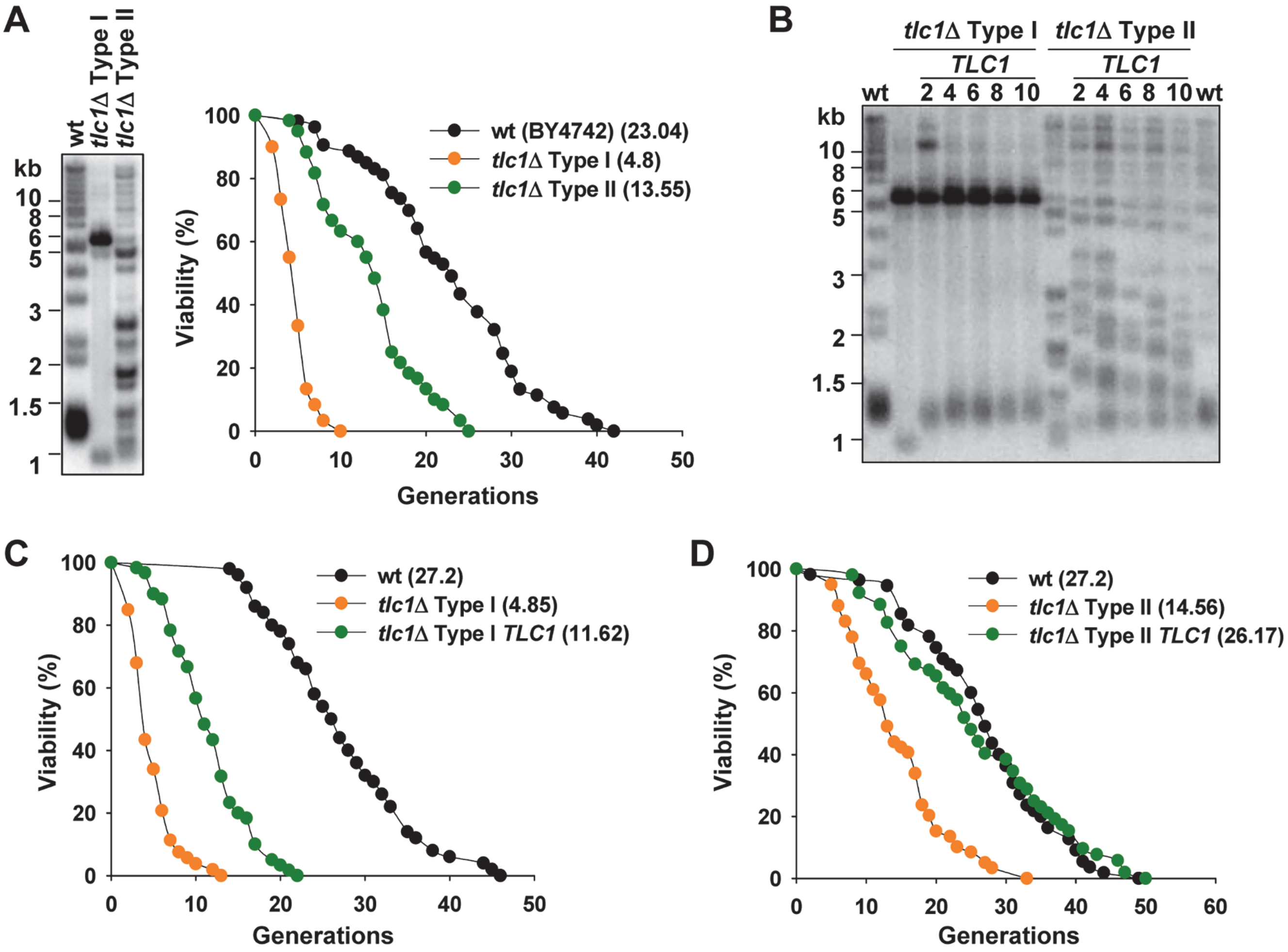

Fig 1. Inactivation of telomerase accelerates yeast replicative aging, and reintroduction of telomerase recovers lifespan. (A) Telomere structure of *tlc1*Δ Type I and Type II survivors were examined by telomere Southern blot (left) and the replicative lifespan of these strains were determined (right). Statistical significance was determined by a Wilcoxon rank sum test and significant differences were stated for $p < 0.05$. The statistical data of all the lifespan experiments in this study were shown in S2 Table. (B) After integration of *TLC1* genes, *tlc1*Δ Type I or Type II strains were continuously passaged and cells at different time point were subjected to telomere Southern blot analyses. The numbers above each lane indicate the numbers of restreaks after *TLC1* reintroduction. (C) and (D) Lifespan analysis of *tlc1*Δ Type I (C) or Type II (D) survivor cells at the 10th restreak after *TLC1* reintroduction.

doi:10.1371/journal.pgen.1005071.g001

Re-activation of telomerase in telomerase-null survivors inhibits telomere recombination and at least partially restores cellular lifespan

The shorter lifespan in telomerase-null survivors suggests that telomere recombination elicits genome instability in telomerase-null survivors to accelerate cellular aging. To test this hypothesis, we re-introduced *TLC1* gene back into the survivors that were derived from *tlc1*Δ cells, by integrating an intact copy of this gene into the genome. After serial passages, telomere structures were examined. The Southern blotting results showed that replenishment of telomerase activity leads to elongation of terminal telomeric TG_{1-3} tracts in Type I cells and the telomere pattern is stably maintained without further Y' amplification (Fig. 1B). On the other hand, reduction of the heterogeneity of the long telomeres was observed in Type II cells after replenishment of *TLC1*, and the telomere structure was gradually restored to a wild-type pattern (Fig. 1B). These results indicate that the recombination activity on telomeres is inhibited by telomerase. Accordingly, reactivation of telomerase partially and completely restores the lifespan

of Type I and Type II cells, respectively (Fig. 1C and 1D). These results support the notion that telomere recombination results in genome instability which causes shorter replicative lifespan. Notably, re-introduction of *TLC1* only partially restored lifespan of Type I survivors (Fig. 1C). This phenotype is likely attributed to the abnormal karyotypes resulting from telomere end-to-end fusions during Type I survivor generation [38,41]. This explanation is supported by the observation that the severe growth defect of Type I survivors was partially recovered after re-introduction of *TLC1*.

Y' element rearrangement mediated by HR occurs in telomerase-positive cells

In telomerase-proficient cells telomere recombination is largely inhibited. However, previous studies have indicated that telomerase seems not to be able to completely eliminate telomere recombination. For example, recombination-mediated telomere rapid deletion (TRD) has been observed in telomerase positive cells [42]. In mouse cells lacking the amino-terminal basic domain of TRF2, t-loop-sized telomeric circles can be excised from leading strand telomeres via homologous recombination [43]. Additionally, homologous recombination occurs with the same frequency in human telomerase-positive and telomerase-negative ALT (alternative lengthening of telomeres) cells [44]. It is conceivable that homologous recombination is intermittently competing with telomerase to contribute to telomere elongation. Due to the repetitive nature of telomeric DNA sequence, telomere recombination products cannot be readily distinguished from those generated by telomerase.

In order to detect telomere recombination event(s) in telomerase-proficient cells, we performed a chromosome healing (*de novo* telomere formation) assay (see Experimental procedures). The system is modified from that developed by Gottschling's lab [45]. Briefly, 81 bp of TG_{1-3} telomeric "seed" (TG81) is inserted into the left arm of chromosome VII at the *ADH4* locus, flanked by a *TRP1* marker gene and an HO endonuclease cutting site (Fig. 2A). Upon HO endonuclease induction by galactose, the HO site is cut and the TG_{1-3} sequence is exposed to the very end. The newly formed telomere of 81 bp is critically short and has to be repaired through telomerase or recombination pathways. As a control, an isogenic strain with no TG_{1-3} seed imbedded (TG0) was included in the experiment. HO-cut will generate a none-telomeric DNA double strand break, which must be repaired to maintain cell viability. In order to examine the repair efficiency of the end generated by HO-cut, we cultured the TG81 or TG0 cells in galactose-containing solid medium, and colonies in which the short telomeres (in TG81 strains) or the DSBs (TG0) have presumably been repaired were counted. The repair efficiency was defined by the number of colonies formed on the galactose plate (cut) divided by that of the same strain on the glucose plate (uncut). The *de novo* generated short telomere can be efficiently repaired with an efficiency of ~100% in the TG81 strain (Fig. 2B). Notably, the efficiency was reduced to ~10% by deletion of *TLC1* (Fig. 2B), suggesting a crucial role of telomerase in the elongation of this new short telomere. Deletion of *RAD50*, *RAD51* or *RAD52* resulted in no or slight reduction in the repair efficiency (Fig. 2B), supporting a predominant role of telomerase rather than recombination in the repairing process. In contrast, the none-telomeric control TG0 strain has very low repair efficiency (~0.36%) (Fig. 2B). Deletion of *TLC1* in the TG0 strain has little effect on the repair efficiency (Fig. 2B), consistent with the notion that the regular DSB generated by HO-cut can hardly be repaired by telomerase. These data indicate that HO-induced double strand break in TG81 strain can generate a bona fide new telomere, which can be readily elongated by telomerase while recombination could still make a minor contribution to its repair.

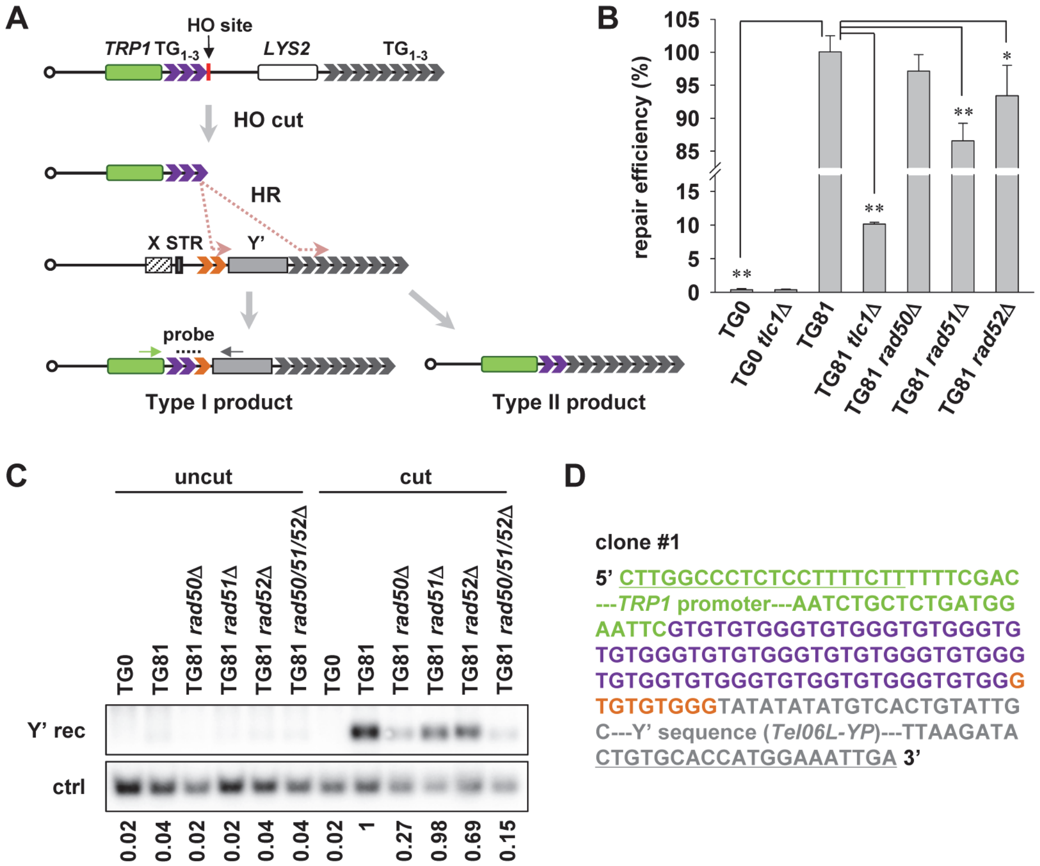

Fig 2. Subtelomeric Y' element recombination occurs in the presence of telomerase. (A) Schematic representation of the strategy to detect Y' recombination in the presence of telomerase (not to scale). 81 bp of TG_{1-3} seed (in purple) is inserted at the *ADH4* gene locus on chromosome VII, flanked by a *TRP1* marker gene (in green) and an HO endonuclease cutting site. The *LYS2* gene was placed between the natural telomere of VII-L and the *ADH4* locus to serve as a genetic marker to monitor HO cutting. After HO induction by galactose, a short telomere with 81 bp of TG_{1-3} sequence is generated *de novo* through HR activity in two distinct regions of the donor telomere: one in TG_{1-3} tracts (in orange) between the STR and the Y' element (Type I) and the other in the terminal TG_{1-3} tracts (Type II). The telomerase-mediated elongation is omitted in this diagram. The resulting Type I recombination products can be detected by PCR amplification using primers specific for *TRP1* (indicated by a green arrow) and Y' consensus sequences (indicated by gray arrow) followed by Southern blot with probes hybridized to TG_{1-3} repeats (indicated by dashes). (B) Cell viability assay for chromosome healing. Proportional yeast cells were plated onto galactose (cut) or glucose-containing medium (uncut). The numbers of colonies formed on the plates were counted and repair efficiency was calculated by dividing the number of colonies on "cut" plates by that on "uncut" plates. The error bars indicates the standard deviations. $*p < 0.05$ and $**p < 0.01$. (C) Southern blot detection of Y' recombination. After HO induction by galactose in liquid culture for 24 h, the isogenic strains (labeled on the top) were subjected to genomic DNA extraction (cut). The uninduced strains were also included in the assay as "uncut" controls. PCR was then performed using primers indicated in A. The PCR products were then subjected to Southern blot with probes recognizing TG_{1-3} sequence (Y' rec). As the loading control, the proportional genomic DNA was digested with EcoRI endonuclease to generate a DNA fragment of 1 kb containing the *POL1* gene sequence and subjected to Southern blot using a probe specific for *POL1* sequence (ctrl). The DNA signals were quantified by software (Multi Gauge). The number below each lane indicates the Y' recombination efficiency, which is defined by dividing the intensity of signal of Y' recombination with that of the internal control. The efficiency of all the samples were compared to that of TG81 cut sample which was set as "1". (D) A representative sequence of one of the three clones showing the sequence of Y' recombination products. Part of the *TRP1* promoter (in green), 78 bp of TG_{1-3} seed sequence (in purple), 9 bp of recombined TG_{1-3} sequence (in orange) and part of the Y' sequence from telomere VIL (*Tel06L-YP*, in gray) are shown. The underlined sequences are primers for PCR as indicated in (A). The full sequence for the PCR product is shown in S1 Fig.

doi:10.1371/journal.pgen.1005071.g002

Next, we used this system to detect whether telomere recombination takes place in telomerase-positive cells. Although it is technically difficult to distinguish the telomeric tracts added by terminal TG_{1-3} recombination (Type II recombination) from those by telomerase, the addition of Y' element to the end of this telomere (Type I recombination) can be detected by Southern blot following PCR amplification with primers specific for *TRP1* region and Y' consensus sequence (Fig. 2A). In this set of experiments, cells were harvested after induction with galactose for 24 h, and genomic DNA was extracted. PCR amplifications were performed using the

genomic DNA as templates. The PCR products were subjected to Southern blot with a TG_{1-3} probe. Meanwhile, proportional amount of genomic DNA was hybridized to a *POL1* probe as the internal control. DNA signals in the Southern blot results were quantified and the level of Y' recombination was normalized to the corresponding internal control. The Y' recombination efficiency of all the samples were compared with that of TG81 cut sample which was defined as "1". We successfully detected the telomere recombination events in TG81 but not TG0 strain by Southern blot (Fig. 2C). To validate that the PCR-amplified fragments contained Y'-sequence, we cloned and sequenced some of the PCR products. The representative sequences of three clones are shown (Fig. 2D and S1 Fig.). As expected, the sequences of the PCR products contain part of the *TRP1* promoter sequence (in green color), variable lengths (87 to 271 bp) of TG_{1-3} repeats (purple for TG seed and orange for telomere sequence from the donor chromosome) and the proximal parts of Y' elements (in gray). Notably, sequences of three clones vary at both the length of internal TG_{1-3} tracts and the origins of Y' elements. Two of the three clones captured the Y' element from the left telomere of chromosome VI, and the third one copied the Y' element from the right telomere of chromosome VIII. These data indicate that HO-induced short telomeres can be repaired by HR in the presence of telomerase, most likely through break-induced replication (BIR) [46].

We also checked the role of some recombination regulators in telomere recombination in TG81 strain in the presence of telomerase. Interestingly, deletion of *RAD50* results in significantly reduced level of such recombination (Fig. 2C). *RAD51*-null cells have unaffected Y' recombination, and deletion of *RAD52* modestly reduces such recombination (Fig. 2C). Paradoxically, it is generally believed that Rad51 and Rad50 are required for the formation of Type I and Type II survivors respectively, and Rad52 is thought to be essential for virtually all homologous recombination activity [32,47]. It remains elusive why Rad51 is dispensable, or Rad52 plays a minor role for the Y' telomere recombination in the presence of telomerase. This kind of recombination events may occur in a way similar to that of BIR, as it was reported that a *rad51*Δ strain still allows BIR to proceed [48], and the Rad51-independent BIR pathway is largely dependent on another set of recombination genes including *RAD50* and *RAD59* [46]. Nevertheless, these results support the argument that HR takes place in telomerase-positive cells.

Deletion of *CGI121* compromises telomere recombination

Because telomere recombination affects cellular lifespan, we propose that inhibition of telomere recombination will be beneficial to cell longevity. Our previous genetic screenings have shown that the evolutionarily conserved KEOPS complex was required for telomeric TG_{1-3} tracts recombination [35]. Additionally KEOPS complex is likely to be involved in generation of telomeric ssDNA because deletion of either *CGI121* or *BUD32* reduces telomeric ssDNA level in *cdc13-1* mutant and suppresses the temperature sensitivity [33]. We therefore wanted to establish the functional relevance of KEOPS complex between telomere recombination and cellular longevity regulation. We focused our efforts on Cgi121 due to several concerns. (1) Deletion of any of the other four subunits confers severe growth defect [33,34,35,49,50], while deletion of *CGI121* only has minor effect on cell growth. (2) Structural studies indicate that lack of Cgi121 doesn't affect the interactions between other subunits [49]. (3) More importantly, Cgi121 is not required for tRNA modification, but indispensable for both telomere length regulation and recombination [33,35,37], providing us a separation-of-function tool to conduct genetic analyses.

To validate that Cgi121 promotes telomere recombination, we firstly examined telomere recombination efficiency of *cgi121*Δ mutant in the presence of telomerase as in Fig. 2C. The result

showed that the Y' recombination efficiency was modestly reduced by deletion of *CGI121* in telomerase-positive cells (Fig. 3A). Then we performed telomere sequencing to examine the role of Cgi121 in telomeric TG_{1-3} recombination in telomerase-negative *tlc1*Δ and *tlc1*Δ *cgi121*Δ cells.

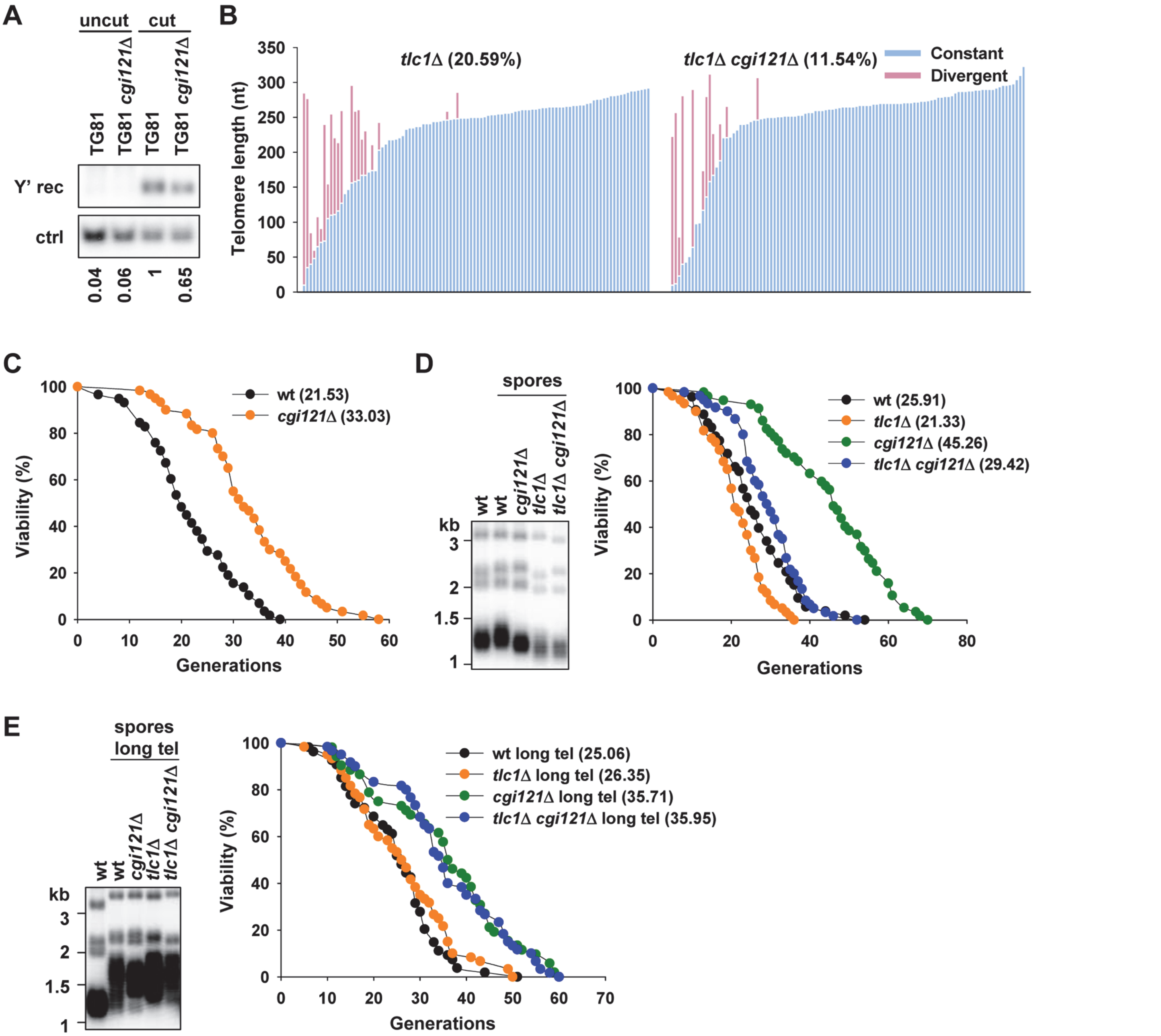

Fig 3. Deletion of KEOPS subunit gene *CGI121* compromises telomere recombination and inhibits cellular aging. (A) Y' recombination of *cgi121*Δ cells. The assay was performed as in Fig. 2C. (B) Telomere sequencing results of *tlc1*Δ and *tlc1*Δ *cgi121*Δ cells. Spores with genotype of *tlc1*Δ and *tlc1*Δ *cgi121*Δ were obtained from the same tetrad and grown for 50 generations before genomic DNA extraction. PCR of telomere IL was performed and the PCR products were ligated to pMD18-T vector and subjected to sequencing. Each column represents one sequenced telomere. The constant parts of the telomere sequences were indicated in blue and the divergent parts in pink (representing the recombined telomere sequences). About 100 clones of each strain were analyzed. (C) Lifespan assay of BY4742 *cgi121*Δ mutant strain. (D) Telomeric Southern blot and lifespan assay of *tlc1*Δ *cgi121*Δ pre-senescing cells. Spores with different genotypes and the same mating type α were grown for 50 generations and then subjected to telomere Southern blot (left) and lifespan analysis (right). (E) After super-elongation of telomeres, telomere length and lifespan of spores with different genotypes and mating type α were determined as in (D).

doi:10.1371/journal.pgen.1005071.g003

We constructed heterozygous diploid cells with one copy of telomerase RNA gene *TLC1* and *CGI121* deleted (BY4743 *TLC1*/ *tlc1*Δ *CGI121*/*cgi121*Δ). After sporulation and tetrad dissection, spores with different genotypes (*tlc1*Δ and *tlc1*Δ *cgi121*Δ) were identified. Spores from the same tetrad were used for further analysis as they had the same initial telomere lengths. Cells were cultured for 50 generations after tetrad dissection and genomic DNA was extracted. Telomere PCR was then performed as previously described to amplify the TG_{1-3} sequence of telomere IL [51,52]. The PCR products were then cloned to T vector for sequencing analyses. About 100 clones of each genotype were obtained and analyzed. The results showed that 20.59% of telomeres (21 out of 102) were elongated through recombination in *tlc1*Δ cells (Fig. 3B). In contrast, only 11.54% of telomeres (12 out of 104) were repaired through recombination in *tlc1*Δ *cgi121*Δ cells (Fig. 3B). These results confirmed that Cgi121 plays a positive regulatory role in telomere recombination in both telomere-positive and -negative cells [35].

Inactivation of Cgi121 promotes cell longevity

Since Cgi121 promotes telomere recombination (Fig. 3A and 3B), and inhibition of telomere recombination restores cellular lifespan (Fig. 1C and 1D), we reasoned that deletion of *CGI121* would suppress the recombination activity at telomeres, and thereby extend the replicative lifespan. Following this thought, we deleted *CGI121* in a long lived yeast strain BY4742 (*Mat* α) which is commonly used in aging research and examined lifespan of the mutant. Deletion of *CGI121* slowed down aging process strikingly, both the mean and maximum lifespan of *cgi121*Δ cells increased about 50% (Fig. 3C). The long live phenotype was also observed in the isogenic BY4741 *cgi121*Δ strain of *Mat* a mating type (S2A Fig.), indicating that Cgi121 affects lifespan independently of the mating type. Thus, we identified Cgi121 as a novel longevity regulator.

As we have mentioned above, in wild-type cells telomeres are maintained mainly by telomerase while telomere recombination occasionally occurs and brings the risk of genome instability. Deletion of *CGI121* in these wild-type cells may inhibit telomere recombination and promote genome stability and cell longevity. If this is the case in the presence of telomerase, the activated telomere recombination in the absence of telomerase could also be compromised by deletion of *CGI121*, and extension of lifespan in telomerase–null cells would be expected.

To examine the role of Cgi121 on lifespan of telomerase-null pre-senescing cells, we obtained spores from heterozygous diploid cells (BY4743 *TLC1*/*tlc1*Δ *CGI121*/*cgi121*Δ) by tetrad dissection. Spores with *Mat* α (the same mating type as that of BY4742) were selected to perform the following lifespan assays. According to previously published data in our lab, cells immediately dissected have lifespan similar to that of wild-type cells as telomere recombination is not activated yet [40]. Thus, in our experiment, spores were grown for 50 generations after dissection so that telomeres are modestly shortened and telomere recombination level is elevated (Fig. 3D, left). These cells were then subjected to lifespan assay. As expected, *tlc1*Δ senescing cells show shortened lifespan, and deletion of *CGI121* extends lifespan of *tlc1*Δ mutant (Fig. 3D, right). Consistently, this phenotype is also observed in *est2*Δ mutant (S2B Fig.). We noticed that lifespan of *tlc1*Δ *cgi121*Δ double mutant was not restored to the level seen in the *cgi121*Δ single mutant (Fig. 3D, right). That's likely attributed to the continuous telomere shortening in the double mutant as the lifespan assay progressing. The emerging critically short telomeres can trigger cell cycle arrest and senescence. Therefore, the double mutant doesn't have a full replicative capacity as the *cgi121*Δ single mutant.

To avoid the interference of critically short telomere(s) on lifespan, we generated heterozygous diploid cells with over-elongated telomeres by introducing a plasmid harboring a Cdc13-Est2 fusion protein [53]. Cells were serially passaged and telomeres were examined by

telomeric Southern blot. Expression of the fusion gene conferred super-long telomeres of about 1 kb to the diploid cells (S2C Fig.). Then the plasmid encoding Cdc13-Est2 fusion protein was popped-out by negative selection and tetrad dissection was performed to obtain spores with different genotypes. The super-long telomeres in the dissected spores are about 800 bp (Fig. 3E, left), a length that prevents critically short telomeres from emerging during the lifespan assay. Over-elongating telomere has no effect on lifespan of wild-type cells (S2D Fig.). The *tlc1Δ* mutant with long telomeres shows similar lifespan to that of wild-type cells (Fig. 3E, right), probably because the long telomeres in this mutant result in similar telomere recombination state to that of wild-type strain. Deletion of *CGI121* extends lifespan of *tlc1Δ* mutant significantly and the double mutant has lifespan similar to that of *cgi121Δ* single mutant (Fig. 3E, right). These data further support our hypothesis that inhibition of telomere recombination by deletion of *CGI121* promotes cellular longevity.

Deletion of *CGI121* promotes longevity in *yku80-4* mutant

It remains elusive how Cgi121 promotes telomere recombination to affect cell longevity. One possibility is through regulating generation of telomeric ssDNA which is essential for initiation of recombination events. Previous report suggests that Cgi121 functions in generation of telomeric ssDNA, as deletion of *CGI121* inhibits accumulation of telomeric ssDNA in the temperature sensitive *cdc13-1* mutant [33]. In wild-type cells, the level of telomeric ssDNA is relatively low. In *yku80Δ* cells, telomeres become deprotected and telomeric ssDNA is accumulated [23,25], and accordingly the replicative lifespan is shortened (S3 Fig.) [54]. However, the shortened lifespan of *yku80Δ* mutant is not restored to the length of wild-type cells by deletion of *CGI121* (S3 Fig.). Considering that yKu80 plays multiple roles in DNA damage repair, as well as telomere maintenance, we then used a separate-of-function *yku80*^{tel} allele, *yku80-4*, which retains the ability of DNA end-joining and telomerase activity regulation, but displays severe defects in telomere protection [55]. The *yku80-4* mutant was constructed by integrating a plasmid bearing a *yku80-4* allele into the genome of *yku80Δ* strain. In parallel, the vector plasmid or the plasmid harboring a wild-type copy of *YKU80* was integrated to *yku80Δ* mutant respectively. As the *yku80Δ* null mutant, the *yku80-4* mutant also shows significantly shortened lifespan (Fig. 4A), probably due to accumulated ssDNA [55]. Strikingly, the lifespan of *yku80-4 cgi121Δ* double mutant is fully restored and extended to a level equivalent to that of the *cgi121Δ* single mutant (Fig. 4B). These data support the conclusion that Cgi121 may facilitate ssDNA generation at telomeres [33], and therefore accelerate cellular aging.

Cgi121 and Fob1 independently regulate cellular lifespan

In budding yeast, the rDNA consists of ~150 copies of 9.1 kb rRNA genes, and is highly recombinogenic [56,57,58]. rDNA instability is promoted by Fob1-dependent DNA replication fork stalling which may cause DSBs within the rDNA [59,60,61]. Elimination of *FOB1* gene reduces the rate of rDNA recombination [61], and significantly extends cellular lifespan [62]. In contrast, deletion of *SIR2* gene disrupts the heterochromatin structure of rDNA loci and rDNA recombination level is elevated which confers shortened lifespan [54,63,64,65]. To investigate whether the effect of Cgi121 on cell longevity is attributed to rDNA recombination, we performed a marker loss assay to analyze the rDNA recombination rate [40]. As controls, *sir2Δ* cells show significantly elevated rDNA recombination level while *fob1Δ* cells have very low level of rDNA recombination (Fig. 5A). The rDNA recombination rate in *cgi121Δ* mutant was comparable to that in wild-type cells (Fig. 5A), indicating that Cgi121 is not involved in rDNA recombination. Consistently, the long lifespan of *cgi121Δ* cells could be further extended by deleting *FOB1* (Fig. 5B). These results demonstrate that rDNA recombination and telomere

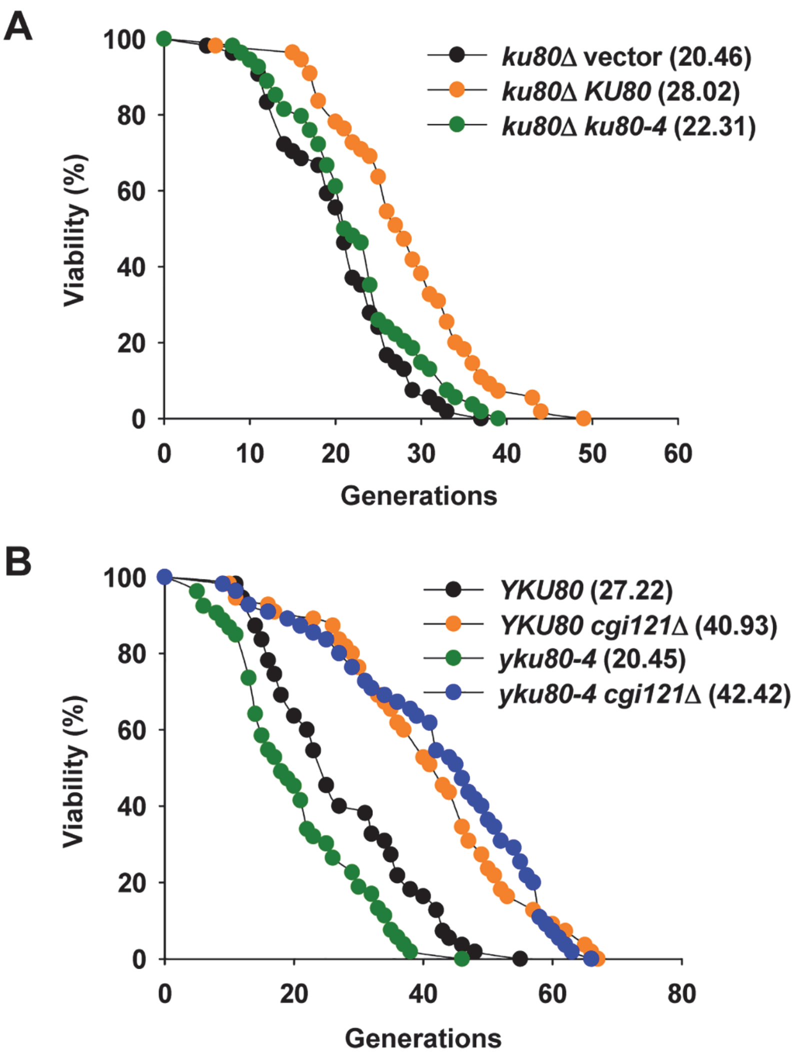

Fig 4. Inactivation of *CGI121* extends lifespan of *yku80-4* cells. (A) Lifespan assay of the *yku80* mutants. (B) Lifespan analysis of *yku80-4* and *yku80-4 cgi121Δ* mutants.

doi:10.1371/journal.pgen.1005071.g004

recombination affect cellular lifespan in different pathways, and inhibition of both recombination activities have additive effect on cell longevity. Interestingly, deletion of Cgi121 affects neither homologous recombination activity at other genomic loci (S4A Fig.) nor the NHEJ efficiency (S4B Fig.), and the gross chromosomal rearrangement (GCR) rate is modestly elevated in *cgi121Δ* mutant (S4C Fig.). Thus, we conclude that the effect of Cgi121 in longevity regulation is attributed specifically to its role in telomere recombination.

Extension of lifespan in *cgi121Δ* cells requires *TOR1*

Calorie restriction (CR) slows aging and increases life span in many organisms [66,67]. The life span extension by CR in yeast is mediated by the coordinated activity of three nutrient-responsive kinases: TOR (target of rapamycin), Sch9, and protein kinase A (PKA) [68,69,70,71]. To better understand the longevity regulation by Cgi121, we examined the lifespan of *cgi121Δ* cells under CR condition. CR treatment was achieved by reducing the glucose concentration in the growth medium from 2% to 0.05% [72]. In this assay, CR treatment extends lifespan of wild-

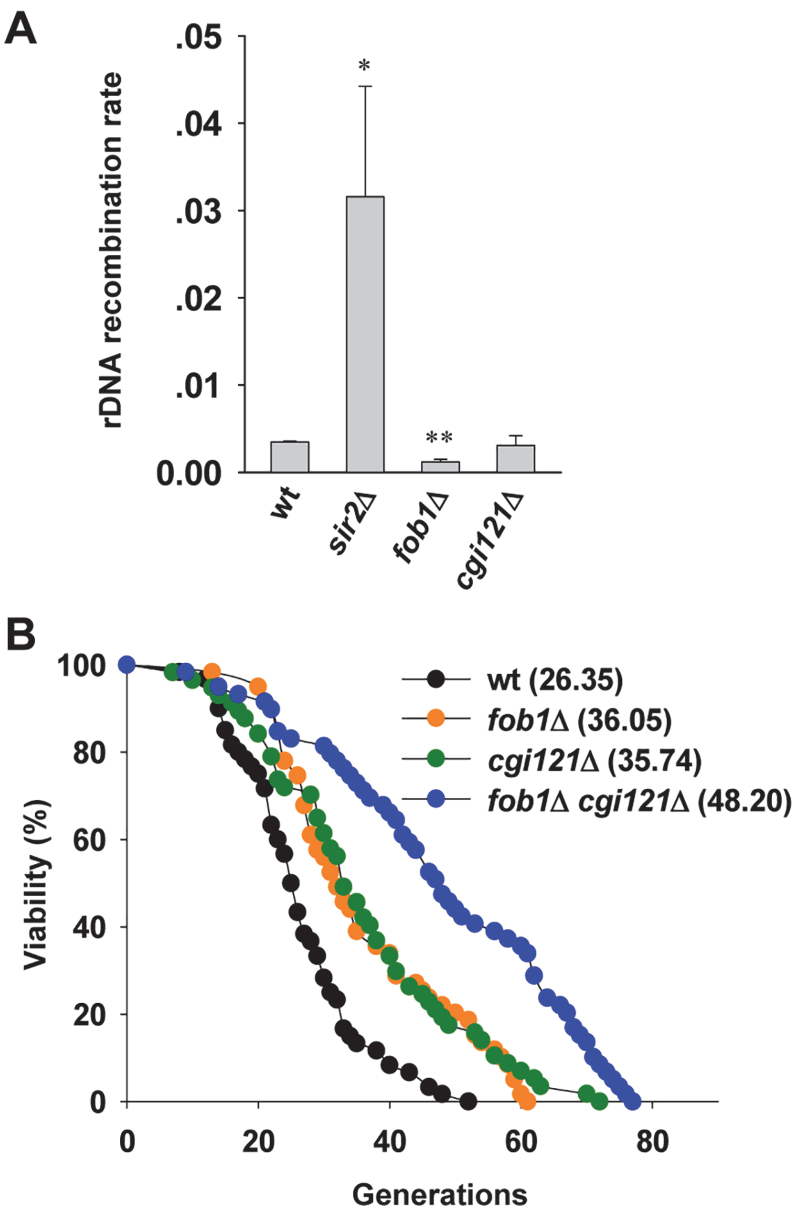

Fig 5. Cgi121 does not affect rDNA recombination and functions in parallel with Fob1 in aging regulation. (A) Examination of rDNA recombination rate in *cgi121*Δ, *fob1*Δ and *sir2*Δ mutants. The error bars indicates the standard deviations. **p* < 0.05 and ***p* < 0.01. (B) Lifespan assay of *cgi121*Δ, *fob1*Δ and *fob1*Δ *cgi121*Δ mutants.

doi:10.1371/journal.pgen.1005071.g005

type cells while the long lifespan of *cgi121*Δ cells could not be maintained under CR condition (Figs. 6A and S5A). Consistently, deletion of *TOR1* which genetically mimics CR [69] shortens the mean and maximum life span of *cgi121*Δ cells (Fig. 6B). The phenotype of lifespan shortening by CR in *cgi121*Δ mutant is quite unexpected, but similar to those observed in W303AR cells, which has been commonly used in yeast aging research [73], as well as in long-lived Osh6 overexpression cells [74].

The observation that the longevity of *cgi121*Δ cells requires TOR activity leads us to speculate that Tor1 might be involved in telomere recombination. To test this possibility, we

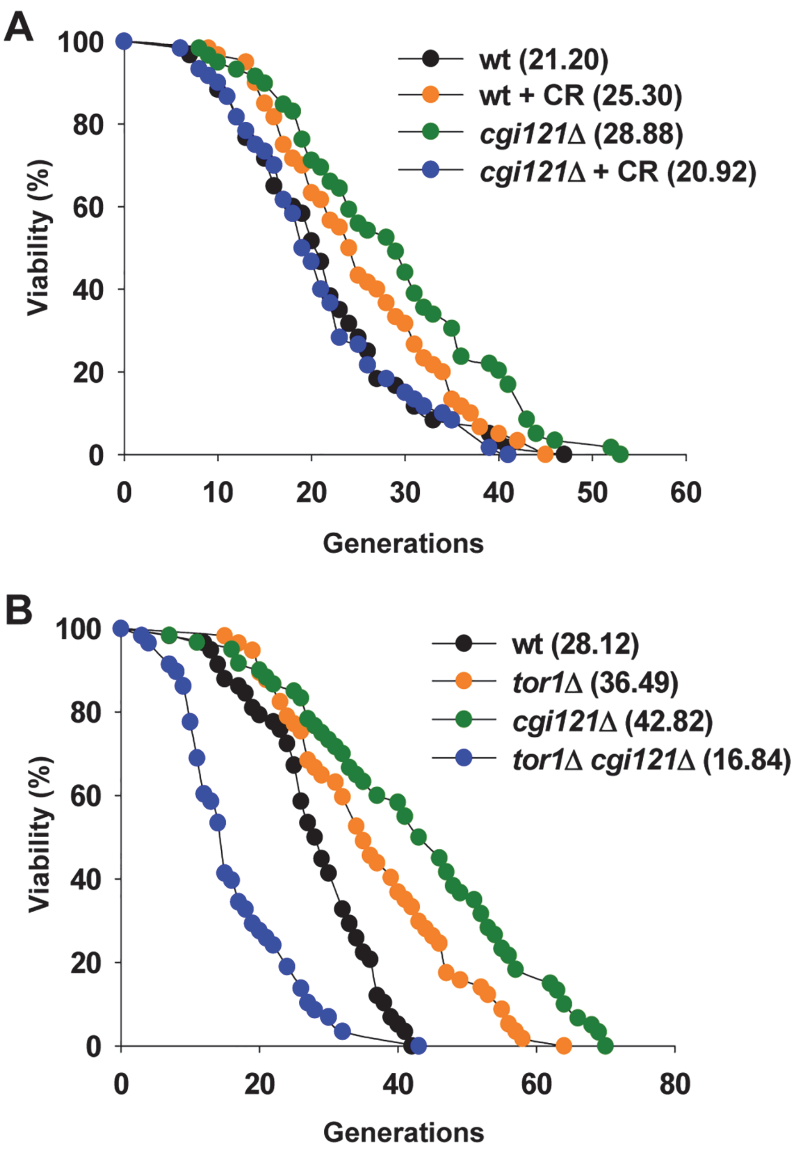

Fig 6. CR shortens lifespan of *cgi121Δ* mutant. (A) Lifespan assay of *cgi121Δ* mutant under CR condition (reducing glucose concentration in the medium from 2% to 0.05%). (B) Lifespan assay of *tor1Δ* and *tor1Δ cgi121Δ* mutants.

doi:10.1371/journal.pgen.1005071.g006

constructed heterozygous diploid cells with one copy of *TOR1* and *TLC1* deleted, and then obtained spores with different genotypes by tetrad dissection. These spores were cultured and serially passaged in solid or liquid medium to see whether deletion of *TOR1* affects either type of telomere recombination. When cultured on solid medium, Type I survivors were readily obtained in *tor1Δ tlc1Δ* cells. 9 out of the 10 randomly selected clones were Type I survivors and the other clone was Type II (S5B Fig.). The emerging frequency of Type I survivors (90%) is highly consistent with previous reports [38,39], suggesting that Y' recombination is unaffected by deletion of *TOR1*. On the other hand, when cultured in liquid medium, *tlc1Δ* cells showed a typical senescence phenotype, and deletion of *TOR1* had no effect on senescence rate according to the growth curve (S5C Fig.). Southern blot analysis revealed that Type II survivors arised in both clones of either *tlc1Δ* or *tor1Δ tlc1Δ* cultures (S5D Fig.), arguing that *TOR1* does not affect

TG recombination. Taken together, these data suggest that Tor1 doesn't affect telomere recombination.

Discussion

Homologous recombination (HR) is generally a universal biological process across the living organisms. It not only serves to eliminate deleterious chromosome lesions (such as DSBs and interstrand crosslinks), but also is critical for the stabilization of stalled replication forks and chromosome segregation in meiosis. Therefore HR is indispensable for general maintenance of genome integrity and stability. Deletion of the genes in *RAD52* epistasis group inhibits telomere recombination, but also results in inability of repairing deleterious lesions inevitably occurring at other chromosomal loci than telomeres. Thus the overall impact of inactivation of *RAD52* epistasis genes on lifespan is negative, leading to genome instability and lifespan shortening [11]. In this work, we surprisingly found that HR activities at telomeres can elicit genome instability and pose a negative effect on cellular longevity. Deletion of the KEOPS subunit gene *CGI121* specifically inhibits telomere recombination and significantly slows down replicative aging (Fig. 3). Cgi121 appears to be only required for HR at telomeres (Fig. 3A and 3B), but not for regular DSB repair by HR at other genomic loci including the rDNA (Figs. 5A and S4A), nor for other DNA repair pathways like NHEJ or GCR (S4B and S4C Fig.). Such a separation-of-function property of Cgi121 provides us a specific tool to assess the effect of telomere recombination in cellular longevity.

Telomerase and HR activities function competitively in telomere maintenance and longevity regulation

Although telomerase-mediated telomere replication is the major pathway that elongates telomeres in most eukaryotes, there are eukaryotes that do not have telomerase, but solely use recombination to maintain telomeres. From the evolutional point of view, HR may represent the earliest telomere maintenance mechanism, which precedes the evolution of telomerase-dependent maintenance of chromosomal termini [75]. In budding yeast, both telomerase and recombination can efficiently function to replicate telomeres, though the former is more preferred. In telomerase-proficient cells, the engagement of recombination in telomere elongation can have both beneficial and detrimental effects (Fig. 7A). On one hand, the recombination activity seems to be complementary to telomerase activity in telomere elongation (Fig. 2). This idea is also supported by the study in the *Kluyveromyces lactis stn1-M1* mutant, in which recombination takes a dominant role in telomere elongation in spite of proficient telomerase activity [76]. Thus it is not surprising, but rather logical, to see that HR is able to function as a back-up system for telomere replication when telomerase pathway fails. On the other hand however, telomeres possess multiple characteristics of DSBs and recruit HR activity to undergo "repair" process. The HR-mediated repair activity tangles or competes with telomerase activity on telomeres (Fig. 2), leading to false-alarms which make the cells undergo aging. Thus, the balance of both activities results in metastable telomeres and normal lifespan (Fig. 7A).

In cells with no or less HR activities at telomeres (Fig. 7B), telomerase is not (or less) "harassed" by the HR activity, and the more stable telomeres result in longer lifespan (Fig. 7B). The KEOPS complex subunit Cgi121 is required for efficient telomere recombination (Fig. 3A), probably by functioning in telomeric ssDNA generation (Fig. 4B) [33]. Deletion of *CGI121* specifically inhibits telomere recombination and confers extended lifespan (Fig. 3).

In telomerase-negative yeast cells, HR becomes the only means to maintain telomere length, and the unstable telomeres elicit genome instability. The trade-off of ensuring the viability of a population of cells through telomere recombination is to partially sacrifice the longevity of the

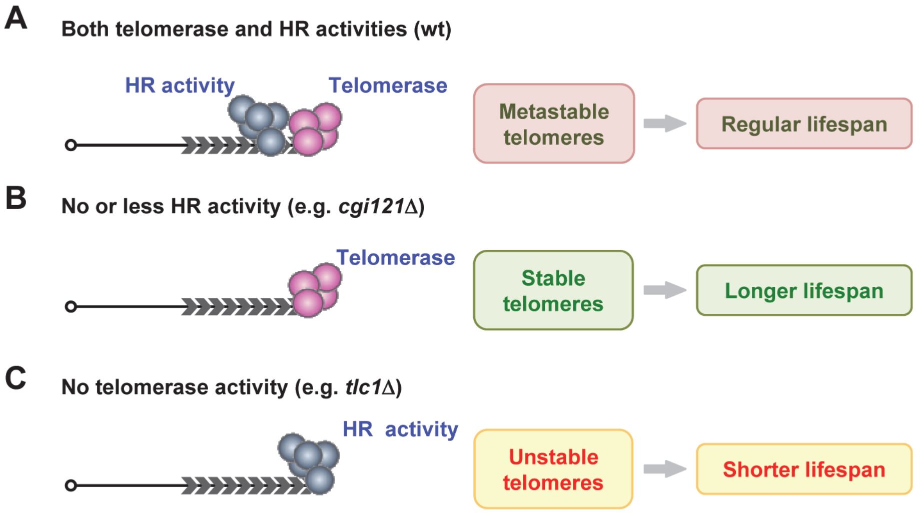

Fig 7. A model of telomere recombination on aging regulation. (A) In wild-type cells, telomerase and HR competes at telomeres to affect cell longevity. (B) In cells that have no or less HR activity (e.g. *cgi121*Δ mutant), telomerase is not disturbed by HR activity. Telomeres are more stable, and results in extended lifespan. (C) In telomerase-deficient cells (e.g. *tlc1*Δ cells), HR activity is the sole force to maintain telomeres, leading to unstable telomeres and shortened lifespan.

doi:10.1371/journal.pgen.1005071.g007

mother cells (Fig. 7C). This notion is supported by several lines of evidence. The yeast cells that solely relied on HR (telomerase-null survivors) exhibited shorter lifespan than those that use telomerase to replicate telomeres (wild-type cells) (Fig. 1A) [40]. Additionally, replenishment of telomerase in telomerase-null yeast cells efficiently inhibits telomere recombination (Fig. 1B), and at least partially restores lifespan (Fig. 1C and 1D). Furthermore, attenuation of telomere recombination by deletion of *CGI121* significantly increases lifespan in telomerase-negative cells (Figs. 3D, 3E and S2B). Therefore, we prefer the model that the illegitimate recombination activity involuntarily competes and interferes with telomerase activity to cause genome instability at telomeres, and results in acceleration of replicative aging (Fig. 7). It is possible that both genome stability and cell longevity are driving forces for the evolution of HR-to-telomerase in telomere maintenance mechanism.

HR activities at different genomic loci have additive effects on aging

HR is prone to take place at the genomic loci that share similar or the same DNA sequences. In yeast genome, both telomere regions and rDNA loci have repetitive sequence, and they are hot spots for intra and/or inter-chromosomal HR. At chromosomal ends, telomere recombination occurs in the presence of telomerase (Fig. 2). Deletion of *CGI121* specifically inhibits telomere recombination and extends cell longevity both in telomerase-positive and -negative cells (Fig. 3). At rDNA loci, the Fob1-dependent replication fork stall causes replication stress and rDNA instability, and triggers recombination-mediated pop-out of rDNA circles [59,60,61]. Deletion of *FOB1* reduces rDNA recombination [61], and extends cellular lifespan [62]. In spite of the differences between telomere and rDNA recombination, inhibition of recombination at both sites seen in the *fob1*Δ *cgi121*Δ double mutant cells has additive effect on slowing down of aging (Fig. 5B). This evidence further supports our model that the unregulated and/or illegitimate HR events occurring at certain genomic loci such as telomeres and rDNA elicit genome instability, and thereby pose a negative impact on cell longevity. It might be

therapeutically significant to find a means to promote cell longevity by blocking recombination-mediated repair of telomeres.

The KEOPS complex and aging regulation

In addition to Cgi121, the KEOPS complex contains four other subunits including a protein kinase (Bud32) and an ATPase (Kae1) [33,34]. It remains elusive whether the other subunits also regulate longevity. Due to the severe growth defect of the other four KEOPS mutants, lifespan assay could not be performed. When a second copy of the KEOPS genes respectively integrated into the genome, the mRNA levels of these genes were elevated respectively according to the qRT-PCR results, suggesting that the five subunits were overexpressed respectively (S6A Fig.). However, the lifespan of the cells overexpressing any of the five subunits was unchanged (S6B and S6C Fig.). We speculate that the subunits of KEOPS complex do not function individually, but rather as a whole complex to regulate aging. Considering the high conservation of KEOPS complex in evolution, it is also intriguing to investigate whether the counterpart of the KEOPS in higher organisms functions in the same way as in yeast in longevity regulation.

Materials and Methods

Yeast strains

All the yeast strains used in this study were listed in S1 Table. Strains used in lifespan assay were all in BY4742 background unless stated otherwise. The *de novo* telomere addition system was modified from that reported by Gottschling's lab [45]. The *yku80-4* and related strains were constructed by integrating an MscI-linearized plasmid pRS303 bearing a copy of *yku80-4* or *YKU80* gene, or simply the vector plasmid into the *his3Δ1* locus in the genome of *yku80Δ* mutant. Strains overexpressing KEOPS subunits were constructed by integrating an MscI-linearized plasmid pRS303 bearing sequences including ORF, endogenous promoter and terminator of the target genes into the *his3Δ1* locus in the genome. Yeast strains were constructed either by transformation with a lithium acetate procedure or genetic cross (mating and tetrad dissection). The plasmids for gene deletion were constructed based on the pRS series [77].

Replicative lifespan assay

Lifespan assay was performed as described previously [40]. Yeast strains were pre-grown overnight on solid YPD plates at 30°C. Cells were then streaked onto fresh YPD plates and grew for about 2 hours. Single cells were randomly selected and arrayed to the plates using a micromanipulator (Singer MSM). After 2 hours (about 1–2 divisions), virgin daughter cells were isolated as buds from mother cells and subjected to lifespan analysis. Daughter cells were then removed by gentle agitation with a dissecting needle and tabulated every 1–2 cell divisions until all the cells stopped dividing. Each experiment was performed with 50–60 cells for each strain. Statistical significance was determined by a Wilcoxon rank sum test using Stata 8 software and significant differences were stated for $p < 0.05$. The statistical data of the replicative lifespan experiments in this study were shown in S2 Table.

Telomere Southern blot

Genomic DNA was extracted and digested with XhoI and then subjected to telomere Southern blot as described previously using the TG_{1-3} probe [52].

Cell viability assay for chromosome healing

Yeast cells were inoculated in yeast complete medium lacking both uracil and lysine ($YC^{U\text{-}K\text{-}}$), containing 2.5% of raffinose (Sigma). The TG81 *rad52Δ* or TG81 *rad50/rad51/rad52Δ* cells were inoculated in YC medium lacking lysine ($YC^{K\text{-}}$). Proportional cells were plated onto YC medium lacking uracil, containing glucose (2%) or galactose (3%) (Sigma). The repair efficiency was defined as the number of colonies on galactose plates (cut) divided by that on glucose plates (uncut). The data were summarized from four independent experimental duplicates and the error bars indicates the standard deviations. Statistic significances were calculated by Student t-test ($^{*}p < 0.05$ and $^{**}p < 0.01$).

Y' recombination detection

Yeast cells were inoculated in $YC^{U\text{-}K\text{-}}$ medium plus 2.5% of raffinose, then diluted into $YC^{U\text{-}}$ plus 2.5% of raffinose and cultured to logarithmic phase. For TG81 *rad52Δ* or TG81 *rad50/rad51/rad52Δ* strain, cells were inoculated in $YC^{K\text{-}}$ medium and diluted into complete YC medium. Galactose was then added to a final concentration of 3%, and cells were cultured for an additional 24 h. Cells were harvested and genomic DNA was extracted following PCR amplification with primers specific for *TRP1* promoter and consensus sequence of all the Y' elements. The PCR products were subjected to Southern blot using a probe specific for TG_{1-3} repeats as used in other telomeric Southern blot in this study. Proportional genomic DNA was digested with EcoRI endonuclease to generate a *POL1* DNA fragment of about 1 kb which was detected by a probe specific for *POL1* gene and serves as internal control. Meanwhile, aliquots of the PCR products were cloned to pMD18-T vector (TAKARA) and subjected to sequencing.

Telomere sequencing

Spores derived from diploid strain BY4743 *TLC1/tlc1Δ CGI121/cgi121Δ* were cultured for about 50 generations after dissection. Genomic DNA was extracted and subjected to telomere PCR as described previously [51,52]. PCR products were cloned to pMD18-T vector (TAKARA) and then subjected to sequencing.

rDNA recombination rate

rDNA recombination rate is assessed by the rate of loss of the *URA3* reporter gene inserted at rDNA loci [40]. Cells grown in log phase were plated to YC medium with or without 0.15% 5-FOA. rDNA recombination rate is determined by dividing the number of colonies grown on 5-FOA-containing YC plates by that on YC plate without 5-FOA. The error bars indicates the standard deviations of data from three independent experiments. Significant differences were stated for $^{*}p < 0.05$ and $^{**}p < 0.01$.

Supporting Information

S1 Fig. The full sequences of the three clones of Y' recombination PCR products in the presence of telomerase. The sequences comprise part of the *TRP1* promoter (in green), variable lengths of TG_{1-3} tracts (including the TG seed in purple and the recombined TG tracts in orange) and the proximal sequences of the Y' elements (in grey). Clone #1 and #2 contain sequence of Y' from telomere VIL (*Tel06L-YP*, in gray), while clone #3 has Y' sequence from telomere VIIIR (*Tel08R-YP*, in grey).
(PDF)

S2 Fig. Deletion of *CGI121* extends lifespan in both telomerase-positive and pre-senescing telomerase-negative cells. (A) BY4741 *cgi121*Δ mutant is subjected to lifespan analysis. (B) Spores with different genotypes and the same mating type α were applied to telomere Southern blot (left) and lifespan analysis (right) as in Fig. 3D. (C) After introduction of a plasmid bearing the *CDC13-EST2* fusion gene or the vector plasmid as a control, the heterozygous diploid cells were continuously passaged and telomere length was examined at the indicated time point by telomeric Southern blot. The numbers above the lanes indicate the numbers of restreaks. (D) Spores of wild-type cells with normal or long telomeres were subjected to telomere length (left) and lifespan analysis (right).
(PDF)

S3 Fig. Deletion of *CGI121* does not affect lifespan of *yku80*Δ mutant. Lifespan of *yku80*Δ and *yku80*Δ *cgi121*Δ mutants was examined.
(PDF)

S4 Fig. Cgi121 does not regulate HR at other genomic loci, NHEJ or GCR efficiency. (A) HR efficiency at other genomic loci was detected for *cgi121*Δ mutant (refer to the "Supporting Materials and Methods" session of S1 Text). The error bars indicates the standard deviations. $^{*}p < 0.05$ and $^{**}p < 0.01$. (B) NHEJ efficiency of *cgi121*Δ mutant. The error bars indicates the standard deviations. $^{*}p < 0.05$ and $^{**}p < 0.01$. (C) GCR efficiency of *cgi121*Δ mutant. The error bars indicates the standard deviations. $^{*}p < 0.05$ and $^{**}p < 0.01$.
(PDF)

S5 Fig. Tor1 does not affect both types of telomere recombination. (A) Lifespan of *cgi121*Δ mutant under different glucose concentration. (B) Southern blot analysis of 10 clones of *tlc1*Δ *tor1*Δ strain obtained by solid medium passage. (C) Growth curve of liquid cultured spores dissected from heterozygous diploid (BY4743 *TLC1/tlc1*Δ *TOR1/tor1*Δ). (D) Southern blot analysis of 2 clones each of *tlc1*Δ and *tlc1*Δ *tor1*Δ cells with liquid medium passage.
(PDF)

S6 Fig. Overexpression of individual KEOPS genes does not affect lifespan. (A) qRT-PCR analysis of the mRNA level of individually overexpressed KEOPS genes. (B) Lifespan analysis of strains overexpressing *BUD32* or *KAE1*. (C) Lifespan analysis of strains overexpressing *CGI121*, *GON7* or *PCC1*.
(PDF)

S1 Table. Yeast strains used in this study.
(PDF)

S2 Table. Statistical data of lifespan experiments in this study.
(PDF)

S1 Text. Supporting materials and methods.
(PDF)

Acknowledgments

We thank Dr. Daniel E. Gottschling (Fred Hutchison Cancer Research Center, USA) and Dr. Hannah L. Klein (New York University School of Medicine, USA) for strains and plasmids, and members of Zhou lab for discussions and suggestions.

Author Contributions

Conceived and designed the experiments: JP YMD YTL JQZ. Performed the experiments: JP MHH. Analyzed the data: JP YTL YMD JQZ. Contributed reagents/materials/analysis tools: JP JQZ. Wrote the paper: JP JQZ.

References

1. Guarente L, Kenyon C (2000) Genetic pathways that regulate ageing in model organisms. Nature 408: 255–262. PMID: 11089983
2. Longo VD, Shadel GS, Kaeberlein M, Kennedy B (2012) Replicative and chronological aging in Saccharomyces cerevisiae. Cell Metab 16: 18–31. doi: 10.1016/j.cmet.2012.06.002 PMID: 22768836
3. Kaeberlein M (2010) Lessons on longevity from budding yeast. Nature 464: 513–519. doi: 10.1038/nature08981 PMID: 20336133
4. Barton AA (1950) Some aspects of cell division in saccharomyces cerevisiae. J Gen Microbiol 4: 84–86. PMID: 15415559
5. Mortimer RK, Johnston JR (1959) Life span of individual yeast cells. Nature 183: 1751–1752. PMID: 13666896
6. Hoeijmakers JH (2009) DNA damage, aging, and cancer. N Engl J Med 361: 1475–1485. doi: 10.1056/NEJMra0804615 PMID: 19812404
7. Lopez-Otin C, Blasco MA, Partridge L, Serrano M, Kroemer G (2013) The hallmarks of aging. Cell 153: 1194–1217. doi: 10.1016/j.cell.2013.05.039 PMID: 23746838
8. McMurray MA, Gottschling DE (2004) Aging and genetic instability in yeast. Curr Opin Microbiol 7: 673–679. PMID: 15556042
9. Burtner CR, Kennedy BK (2010) Progeria syndromes and ageing: what is the connection? Nat Rev Mol Cell Biol 11: 567–578. doi: 10.1038/nrm2944 PMID: 20651707
10. Sinclair DA, Guarente L (1997) Extrachromosomal rDNA circles—a cause of aging in yeast. Cell 91: 1033–1042. PMID: 9428525
11. Park PU, Defossez PA, Guarente L (1999) Effects of mutations in DNA repair genes on formation of ribosomal DNA circles and life span in Saccharomyces cerevisiae. Mol Cell Biol 19: 3848–3856. PMID: 10207108
12. McEachern MJ, Krauskopf A, Blackburn EH (2000) Telomeres and their control. Annu Rev Genet 34: 331–358. PMID: 11092831
13. Wellinger RJ, Wolf AJ, Zakian VA (1993) Saccharomyces telomeres acquire single-strand TG1–3 tails late in S phase. Cell 72: 51–60. PMID: 8422682
14. Wellinger RJ, Zakian VA (2012) Everything you ever wanted to know about Saccharomyces cerevisiae telomeres: beginning to end. Genetics 191: 1073–1105. doi: 10.1534/genetics.111.137851 PMID: 22879408
15. Lingner J, Cech TR, Hughes TR, Lundblad V (1997) Three Ever Shorter Telomere (EST) genes are dispensable for in vitro yeast telomerase activity. Proc Natl Acad Sci U S A 94: 11190–11195. PMID: 9326584
16. Singer MS, Gottschling DE (1994) TLC1: template RNA component of Saccharomyces cerevisiae telomerase. Science 266: 404–409. PMID: 7545955
17. Greider CW, Blackburn EH (1985) Identification of a specific telomere terminal transferase activity in Tetrahymena extracts. Cell 43: 405–413. PMID: 3907856
18. Tong XJ, Li QJ, Duan YM, Liu NN, Zhang ML, et al. (2011) Est1 protects telomeres and inhibits subtelomeric y'-element recombination. Mol Cell Biol 31: 1263–1274. doi: 10.1128/MCB.00831-10 PMID: 21220516
19. Lundblad V, Szostak JW (1989) A mutant with a defect in telomere elongation leads to senescence in yeast. Cell 57: 633–643. PMID: 2655926
20. Lendvay TS, Morris DK, Sah J, Balasubramanian B, Lundblad V (1996) Senescence mutants of Saccharomyces cerevisiae with a defect in telomere replication identify three additional EST genes. Genetics 144: 1399–1412. PMID: 8978029
21. Boulton SJ, Jackson SP (1996) Identification of a Saccharomyces cerevisiae Ku80 homologue: roles in DNA double strand break rejoining and in telomeric maintenance. Nucleic Acids Res 24: 4639–4648. PMID: 8972848
22. Milne GT, Jin S, Shannon KB, Weaver DT (1996) Mutations in two Ku homologs define a DNA end-joining repair pathway in Saccharomyces cerevisiae. Mol Cell Biol 16: 4189–4198. PMID: 8754818

23. Polotnianka RM, Li J, Lustig AJ (1998) The yeast Ku heterodimer is essential for protection of the telomere against nucleolytic and recombinational activities. Curr Biol 8: 831–834. PMID: 9663392
24. Boulton SJ, Jackson SP (1998) Components of the Ku-dependent non-homologous end-joining pathway are involved in telomeric length maintenance and telomeric silencing. EMBO J 17: 1819–1828. PMID: 9501103
25. Gravel S, Larrivee M, Labrecque P, Wellinger RJ (1998) Yeast Ku as a regulator of chromosomal DNA end structure. Science 280: 741–744. PMID: 9563951
26. Fisher TS, Taggart AK, Zakian VA (2004) Cell cycle-dependent regulation of yeast telomerase by Ku. Nat Struct Mol Biol 11: 1198–1205. PMID: 15531893
27. Stellwagen AE, Haimberger ZW, Veatch JR, Gottschling DE (2003) Ku interacts with telomerase RNA to promote telomere addition at native and broken chromosome ends. Genes Dev 17: 2384–2395. PMID: 12975323
28. DuBois ML, Haimberger ZW, McIntosh MW, Gottschling DE (2002) A quantitative assay for telomere protection in Saccharomyces cerevisiae. Genetics 161: 995–1013. PMID: 12136006
29. Nakada D, Hirano Y, Sugimoto K (2004) Requirement of the Mre11 complex and exonuclease 1 for activation of the Mec1 signaling pathway. Mol Cell Biol 24: 10016–10025. PMID: 15509802
30. Mimitou EP, Symington LS (2008) Sae2, Exo1 and Sgs1 collaborate in DNA double-strand break processing. Nature 455: 770–774. doi: 10.1038/nature07312 PMID: 18806779
31. Nakada D, Matsumoto K, Sugimoto K (2003) ATM-related Tel1 associates with double-strand breaks through an Xrs2-dependent mechanism. Genes Dev 17: 1957–1962. PMID: 12923051
32. McEachern MJ, Haber JE (2006) Break-induced replication and recombinational telomere elongation in yeast. Annu Rev Biochem 75: 111–135. PMID: 16756487
33. Downey M, Houlsworth R, Maringele L, Rollie A, Brehme M, et al. (2006) A genome-wide screen identifies the evolutionarily conserved KEOPS complex as a telomere regulator. Cell 124: 1155–1168. PMID: 16564010
34. Kisseleva-Romanova E, Lopreiato R, Baudin-Baillieu A, Rousselle JC, Ilan L, et al. (2006) Yeast homolog of a cancer-testis antigen defines a new transcription complex. EMBO J 25: 3576–3585. PMID: 16874308
35. Hu Y, Tang HB, Liu NN, Tong XJ, Dang W, et al. (2013) Telomerase-null survivor screening identifies novel telomere recombination regulators. PLoS Genet 9: e1003208. doi: 10.1371/journal.pgen.1003208 PMID: 23390378
36. El Yacoubi B, Hatin I, Deutsch C, Kahveci T, Rousset JP, et al. (2011) A role for the universal Kae1/Qri7/YgjD (COG0533) family in tRNA modification. EMBO J 30: 882–893. doi: 10.1038/emboj.2010.363 PMID: 21285948
37. Srinivasan M, Mehta P, Yu Y, Prugar E, Koonin EV, et al. (2011) The highly conserved KEOPS/EKC complex is essential for a universal tRNA modification, t6A. EMBO J 30: 873–881. doi: 10.1038/emboj.2010.343 PMID: 21183954
38. Lundblad V, Blackburn EH (1993) An alternative pathway for yeast telomere maintenance rescues est1- senescence. Cell 73: 347–360. PMID: 8477448
39. Teng SC, Zakian VA (1999) Telomere-telomere recombination is an efficient bypass pathway for telomere maintenance in Saccharomyces cerevisiae. Mol Cell Biol 19: 8083–8093. PMID: 10567534
40. Chen XF, Meng FL, Zhou JQ (2009) Telomere recombination accelerates cellular aging in Saccharomyces cerevisiae. PLoS Genet 5: e1000535. doi: 10.1371/journal.pgen.1000535 PMID: 19557187
41. Liti G, Louis EJ (2003) NEJ1 prevents NHEJ-dependent telomere fusions in yeast without telomerase. Mol Cell 11: 1373–1378. PMID: 12769859
42. Li B, Lustig AJ (1996) A novel mechanism for telomere size control in Saccharomyces cerevisiae. Genes Dev 10: 1310–1326. PMID: 8647430
43. Wang RC, Smogorzewska A, de Lange T (2004) Homologous recombination generates T-loop-sized deletions at human telomeres. Cell 119: 355–368. PMID: 15507207
44. Bechter OE, Zou Y, Shay JW, Wright WE (2003) Homologous recombination in human telomerase-positive and ALT cells occurs with the same frequency. EMBO Rep 4: 1138–1143. PMID: 14618159
45. Diede SJ, Gottschling DE (1999) Telomerase-mediated telomere addition in vivo requires DNA primase and DNA polymerases alpha and delta. Cell 99: 723–733. PMID: 10619426
46. Signon L, Malkova A, Naylor ML, Klein H, Haber JE (2001) Genetic requirements for RAD51- and RAD54-independent break-induced replication repair of a chromosomal double-strand break. Mol Cell Biol 21: 2048–2056. PMID: 11238940
47. Le S, Moore JK, Haber JE, Greider CW (1999) RAD50 and RAD51 define two pathways that collaborate to maintain telomeres in the absence of telomerase. Genetics 152: 143–152. PMID: 10224249

48. Malkova A, Ivanov EL, Haber JE (1996) Double-strand break repair in the absence of RAD51 in yeast: a possible role for break-induced DNA replication. Proc Natl Acad Sci U S A 93: 7131–7136. PMID: 8692957

49. Mao DY, Neculai D, Downey M, Orlicky S, Haffani YZ, et al. (2008) Atomic structure of the KEOPS complex: an ancient protein kinase-containing molecular machine. Mol Cell 32: 259–275. doi: 10.1016/j.molcel.2008.10.002 PMID: 18951093

50. Hecker A, Lopreiato R, Graille M, Collinet B, Forterre P, et al. (2008) Structure of the archaeal Kae1/Bud32 fusion protein MJ1130: a model for the eukaryotic EKC/KEOPS subcomplex. EMBO J 27: 2340–2351. doi: 10.1038/emboj.2008.157 PMID: 19172740

51. Forstemann K, Hoss M, Lingner J (2000) Telomerase-dependent repeat divergence at the 3' ends of yeast telomeres. Nucleic Acids Res 28: 2690–2694. PMID: 10908324

52. Meng FL, Hu Y, Shen N, Tong XJ, Wang J, et al. (2009) Sua5p a single-stranded telomeric DNA-binding protein facilitates telomere replication. EMBO J 28: 1466–1478. doi: 10.1038/emboj.2009.92 PMID: 19369944

53. Evans SK, Lundblad V (1999) Est1 and Cdc13 as comediators of telomerase access. Science 286: 117–120. PMID: 10506558

54. Kaeberlein M, McVey M, Guarente L (1999) The SIR2/3/4 complex and SIR2 alone promote longevity in Saccharomyces cerevisiae by two different mechanisms. Genes Dev 13: 2570–2580. PMID: 10521401

55. Bertuch AA, Lundblad V (2003) The Ku heterodimer performs separable activities at double-strand breaks and chromosome termini. Mol Cell Biol 23: 8202–8215. PMID: 14585978

56. Petes TD, Botstein D (1977) Simple Mendelian inheritance of the reiterated ribosomal DNA of yeast. Proc Natl Acad Sci U S A 74: 5091–5095. PMID: 337310

57. Philippsen P, Thomas M, Kramer RA, Davis RW (1978) Unique arrangement of coding sequences for 5 S, 5.8 S, 18 S and 25 S ribosomal RNA in Saccharomyces cerevisiae as determined by R-loop and hybridization analysis. J Mol Biol 123: 387–404. PMID: 357737

58. Rustchenko EP, Sherman F (1994) Physical constitution of ribosomal genes in common strains of Saccharomyces cerevisiae. Yeast 10: 1157–1171. PMID: 7754705

59. Kobayashi T, Horiuchi T (1996) A yeast gene product, Fob1 protein, required for both replication fork blocking and recombinational hotspot activities. Genes Cells 1: 465–474. PMID: 9078378

60. Johzuka K, Horiuchi T (2002) Replication fork block protein, Fob1, acts as an rDNA region specific recombinator in S. cerevisiae. Genes Cells 7: 99–113. PMID: 11895475

61. Kobayashi T, Heck DJ, Nomura M, Horiuchi T (1998) Expansion and contraction of ribosomal DNA repeats in Saccharomyces cerevisiae: requirement of replication fork blocking (Fob1) protein and the role of RNA polymerase I. Genes Dev 12: 3821–3830. PMID: 9869636

62. Defossez PA, Prusty R, Kaeberlein M, Lin SJ, Ferrigno P, et al. (1999) Elimination of replication block protein Fob1 extends the life span of yeast mother cells. Mol Cell 3: 447–455. PMID: 10230397

63. Bryk M, Banerjee M, Murphy M, Knudsen KE, Garfinkel DJ, et al. (1997) Transcriptional silencing of Ty1 elements in the RDN1 locus of yeast. Genes Dev 11: 255–269. PMID: 9009207

64. Smith JS, Boeke JD (1997) An unusual form of transcriptional silencing in yeast ribosomal DNA. Genes Dev 11: 241–254. PMID: 9009206

65. Gottlieb S, Esposito RE (1989) A new role for a yeast transcriptional silencer gene, SIR2, in regulation of recombination in ribosomal DNA. Cell 56: 771–776. PMID: 2647300

66. Anderson RM, Weindruch R (2012) The caloric restriction paradigm: implications for healthy human aging. Am J Hum Biol 24: 101–106. doi: 10.1002/ajhb.22243 PMID: 22290875

67. Katewa SD, Kapahi P (2010) Dietary restriction and aging, 2009. Aging Cell 9: 105–112. doi: 10.1111/j.1474-9726.2010.00552.x PMID: 20096035

68. Fabrizio P, Pozza F, Pletcher SD, Gendron CM, Longo VD (2001) Regulation of longevity and stress resistance by Sch9 in yeast. Science 292: 288–290. PMID: 11292860

69. Kaeberlein M, Powers RW 3rd, Steffen KK, Westman EA, Hu D, et al. (2005) Regulation of yeast replicative life span by TOR and Sch9 in response to nutrients. Science 310: 1193–1196. PMID: 16293764

70. Lin SJ, Defossez PA, Guarente L (2000) Requirement of NAD and SIR2 for life-span extension by calorie restriction in Saccharomyces cerevisiae. Science 289: 2126–2128. PMID: 11000115

71. Powers RW 3rd, Kaeberlein M, Caldwell SD, Kennedy BK, Fields S (2006) Extension of chronological life span in yeast by decreased TOR pathway signaling. Genes Dev 20: 174–184. PMID: 16418483

72. Kaeberlein M, Kirkland KT, Fields S, Kennedy BK (2005) Genes determining yeast replicative life span in a long-lived genetic background. Mech Ageing Dev 126: 491–504. PMID: 15722108

73. Kaeberlein M, Steffen KK, Hu D, Dang N, Kerr EO, et al. (2006) Comment on "HST2 mediates SIR2-independent life-span extension by calorie restriction". Science 312: 1312; author reply 1312. PMID: 16741098

74. Gebre S, Connor R, Xia Y, Jawed S, Bush JM, et al. (2012) Osh6 overexpression extends the lifespan of yeast by increasing vacuole fusion. Cell Cycle 11: 2176–2188. doi: 10.4161/cc.20691 PMID: 22622083

75. de Lange T (2004) T-loops and the origin of telomeres. Nat Rev Mol Cell Biol 5: 323–329. PMID: 15071557

76. Iyer S, Chadha AD, McEachern MJ (2005) A mutation in the STN1 gene triggers an alternative lengthening of telomere-like runaway recombinational telomere elongation and rapid deletion in yeast. Mol Cell Biol 25: 8064–8073. PMID: 16135798

77. Sikorski RS, Hieter P (1989) A system of shuttle vectors and yeast host strains designed for efficient manipulation of DNA in Saccharomyces cerevisiae. Genetics 122: 19–27. PMID: 2659436
